# Dynamically regulated focal adhesions coordinate endothelial cell remodelling in developing vasculature

**DOI:** 10.1242/dev.200454

**Published:** 2022-12-12

**Authors:** Tevin C. Y. Chau, Mikaela S. Keyser, Jason A. Da Silva, Elysse K. Morris, Teodor E. Yordanov, Kinga P. Duscyz, Scott Paterson, Alpha S. Yap, Benjamin M. Hogan, Anne Karine Lagendijk

**Affiliations:** ^1^ Division of Cell and Developmental Biology, Institute for Molecular Bioscience, The University of Queensland, St Lucia, Queensland 4072, Australia; ^2^ Organogenesis and Cancer Program, Peter MacCallum Cancer Centre and The PeterMac Callum Department of Oncology, The University of Melbourne, Melbourne, Victoria 3000, Australia; ^3^ Department of Anatomy and Physiology, The University of Melbourne, Parkville, Victoria 3010, Australia; ^4^ School of Biomedical Sciences, The University of Queensland, Brisbane, Queensland 4072, Australia

**Keywords:** Talin1, Vinculin, Focal adhesions, Endothelial cell remodelling, Cell-cell junctions, Zebrafish

## Abstract

The assembly of a mature vascular network involves coordinated endothelial cell (EC) shape changes, including the process of EC elongation. How EC elongation is dynamically regulated *in vivo* is not fully understood. Here, we have generated a zebrafish mutant that is deficient for the integrin adaptor protein Talin 1 (Tln1). Using a new focal adhesion (FA) marker line expressing endothelial Vinculinb-eGFP, we demonstrate that EC FAs function dynamically and are lost in our *tln1* mutants, allowing us to uncouple the primary roles of FAs in EC morphogenesis from the secondary effects that occur due to systemic vessel failure or loss of blood flow. Tln1 loss led to compromised F-actin rearrangements, perturbed EC elongation and disrupted cell-cell junction linearisation in vessel remodelling. Finally, chemical induction of actin polymerisation restored actin dynamics and EC elongation during vascular morphogenesis. Together, we identify that FAs are essential for EC elongation and junction linearisation in flow-pressured vessels and that they influence actin polymerisation in cellular morphogenesis. These observations can explain the severely compromised vessel beds and vascular leakage observed in mutant models that lack integrin signalling.

This article has an associated ‘The people behind the papers’ interview.

## Teaser

**Highlighted Article:** Focal adhesions drive actin rearrangements that are essential for endothelial cell elongation and junction linearisation in flow-pressured vessels.

## INTRODUCTION

Cells are mechanically linked to the extracellular matrix (ECM) via dynamic protein complexes, whereby integrin heterodimers form the main ECM ligand-binding receptors that couple adhesion to the actin cytoskeleton (Kechagia et al., 2019; Sun et al., 2016). Integrins, however, cannot bind to actin directly, and full mechanical linkage is achieved via adaptor proteins such as talins (Calderwood et al., 2013; Goult et al., 2018; Kechagia et al., 2019; Klapholz and Brown, 2017; Sun et al., 2016). Talins are recruited to ligand-bound integrins; mechanical tension applied externally or from the actin cytoskeleton can unfold talins to reveal additional binding sites for other proteins, such as vinculin (Goult et al., 2018). Via this direct and indirect recruitment of numerous cytosolic proteins, cell-ECM adhesion structures are formed that control both biochemical and biophysical signalling. Further clustering of integrins, actin bundling and re-enforcement of these interactions lead to the maturation of cell-ECM adhesion sites, referred to as focal adhesions (FAs) (Harburger and Calderwood, 2009). Talins are required for FA formation, actin attachment and FA maturation (Goult et al., 2010, 2018). Importantly, FAs do not exist and function as isolated adhesive structures, instead significant crosstalk occurs with cadherin-based adhesions at cell-cell junctions (Mui et al., 2016; Schwartz and DeSimone, 2008; Weber et al., 2011). Crosstalk happens by reciprocal binding to the actin cytoskeleton and via recruitment of identical intracellular effector proteins; thus, the FAs and the adherens junctions together coordinate a cells adhesive properties, morphogenesis and mechanical state.

Since the initial discoveries of cardiovascular defects in mouse models with compromised integrin signalling (Rupp and Little, 2001), vascular biologists have had a great interest in understanding how FAs alter vessel growth, function and stability. At the receptor level, the integrin-β1 subunit, as well as the α5/αv subunits, have been proven to be essential for angiogenesis and vascular integrity (Dejana et al., 1990; Malinin et al., 2012; van der Flier et al., 2010; Wu et al., 2001). Underpinning the relevance of talins to integrin function is the observation that endothelial specific knockout (KO) of talin 1 (Tln1) (the main talin protein expressed by ECs; Kopp et al., 2010; Monkley et al., 2011) results in similar phenotypes to integrin loss of function (Monkley et al., 2011; Pulous et al., 2021), including reduced vessel network complexity and haemorrhaging. Likewise,Tln1-deficient zebrafish develop haemorrhages in the brain; however, vascular morphogenesis has not been analysed in detail in this zebrafish model (Wu et al., 2015). Reflecting the known crosstalk between FAs and adherens junctions, haemorrhaging of FA-deficient vasculature has therefore been suggested to be a consequence of impaired crosstalk, as disrupted VE-cadherin localisation has been identified in the retinal vasculature of integrin β1 and *Tln1* KO mice (Pulous et al., 2021; Yamamoto et al., 2015). However, the mechanisms underlying this crosstalk remain to be determined.

Although landmark studies using KO mice have clearly shown that FAs are essential for vascular morphogenesis and vessel function, constitutive EC KOs developed severe cardiac and vascular abnormalities that prohibited analysis of functional vessels under flow (Carlson et al., 2008; Lei et al., 2008; Monkley et al., 2011; Tanjore et al., 2008; Zovein et al., 2010). It is well appreciated that FA assembly and function in ECs is dependent on cell extrinsic factors imposed by blood flow and flow-dependent shear stress (Chen et al., 1999; Orr et al., 2006; Tzima et al., 2001, 2005). The severity of the mouse KO phenotypes might therefore have complicated the identification of primary cellular processes that require FA function during early stages of vascular network formation.

In this study, we sought to better understand the early vascular impact of FAs, using a novel zebrafish *tln1* mutant model, *tln1^uq1al^*. In *tln1^uq1al−/−^* mutants, cardiac output remains stable, and thus blood circulation persists, until 2 days post fertilisation (2 dpf), providing a developmental time window to study early vascular events when blood flow is preserved. In other words, this model provides a unique opportunity to study the EC-specific consequences of compromised FA function in flow pressured vasculature of live animals. Using a novel FA marker line that expresses a vascular restricted Vinculinb-eGFP, we show that Tln1 loss leads to a lack of mature, Vinculinb-positive, FAs. High-resolution live-imaging of VE-cadherin and F-actin uncovered that FAs are required for EC elongation and cell-cell junction linearisation. Mechanistically, enhancing actin polymerisation restores EC elongation and junction linearisation of FA-deficient ECs. Overall, this work has identified that FAs are required for vessel remodelling by driving actin rearrangements and that these rearrangements are translated into EC elongation and linearisation of EC junctions during vascular morphogenesis. These defects in EC elongation and junction linearisation likely contribute to the severely compromised vessel beds, haemorrhage and vascular leakage observed in the absence of vascular integrin signalling.

## RESULTS

### Establishing *in vivo* models to study focal adhesion function and dynamics during blood vessel morphogenesis

**Fig. 1.**
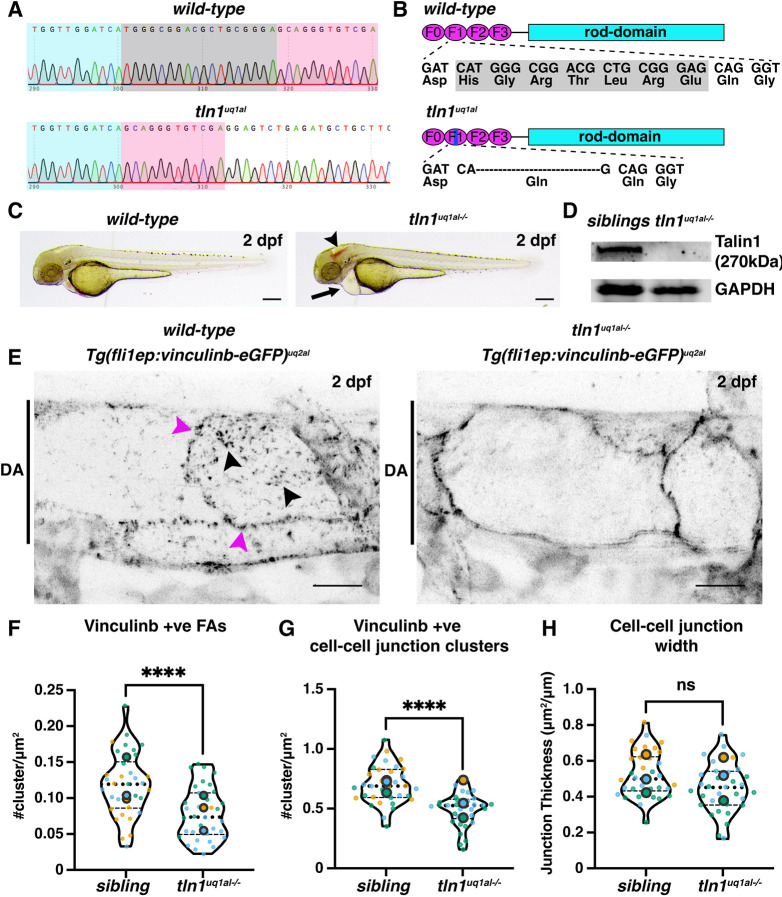
**Novel zebrafish models for studying focal adhesion function and dynamics in live vasculature.** (A) Chromatograms of genomic sequencing of a wild-type sibling embryo (top) and a homozygous *tln1^uq1al−/−^* mutant (bottom). Bases highlighted in grey in the wild type are deleted in *tln1^uq1al−/−^* mutants. (B) Schematics of Talin 1 protein showing the 18 bp deletion in *tln1^uq1al−/−^* resulting in an in-frame deletion of six amino acids (His-Gly-Arg-Thr-Leu-Arg) and a Glu-to-Gln substitution in the F1 FERM domain. (C) Bright-field images showing body morphology of a wild-type sibling versus a *tln1^uq1al−/−^* mutant at 2 dpf. Arrowhead indicates cranial haemorrhaging in the mutant. Arrow indicates cardiac oedema in the mutant. Scale bars: 100 µm. (D) Western blot analysis at 3 dpf showing loss of Talin 1 in *tln1^uq1al−/−^* mutants. (E) Expression of Vinculinb-eGFP at FAs (black arrowheads) and at cell-cell junctions (magenta arrowheads) in the dorsal aorta (DA) of a wild-type sibling at 2 dpf (left). In *tln1^uq1al−/−^* mutants (right), expression at FAs in greatly reduced, while a diffuse presence of Vinculinb-eGFP is observed at cell-cell junctions. Scale bars: 10 µm. (F) Number of Vinculinb-eGFP-positive FA clusters, *n*=3 replicates: *n*=23 ECs siblings and *n*=18 ECs *tln1^uq1al−/−^* mutants (****P<0.0001, unpaired *t*-test ). (G) Number of Vinculinb-eGFP-positive clusters at cell-cell junctions, *n*=3 replicates: *n*=23 ECs siblings and *n*=18 ECs *tln1^uq1al−/−^* mutants (****P<0.0001, unpaired *t*-test ). (H) Quantification of cell-cell junction width based on Vinculin-eGFP expression, *n*=3 replicates: *n*=23 ECs siblings and *n*=18 ECs *tln1^uq1al−/−^* mutants, no significant difference (ns) (unpaired *t*-test). Replicate averages are depicted by large circles; smaller circles indicate individual data points of each replicate (colour matched).

To study the role of cell-ECM coupling in vascular morphogenesis, we used CRISPR/Cas9 genome editing to generate a loss-of-function model for Tln1 in zebrafish. We targeted Tln1 as this is the main protein that links activated integrins to the actin cytoskeleton and is essential for FA formation and downstream signalling (Calderwood et al., 2013; Goult et al., 2018; Klapholz and Brown, 2017). The resultant *tln1^uq1al^* allele harbours an in-frame deletion of six amino acids and substitution of one amino acid in the F1 FERM-domain, which is essential for integrin binding (Calderwood et al., 2013; Goult et al., 2018) (Fig. 1A,B and Fig. S1A). Phenotypically, the first time-point at which we could distinguish *tln1^uq1al−/−^* mutants from siblings was at 2 days post fertilisation (2 dpf). The mutants present with cranial haemorrhaging, caused by a severely compromised cranial vessel network (Fig. 1C and Fig. S1B). This haemorrhaging phenotype is comparable with those observed in other mice and zebrafish knockout models for FA components (Carlson et al., 2008; Iida et al., 2018; Lei et al., 2008; Monkley et al., 2011; Pulous et al., 2021, 2019; Tanjore et al., 2008; Wu et al., 2015; Yamamoto et al., 2015; Zovein et al., 2010). Based on the predicted structure and location of the missing amino acids using Alphafold2 (Jumper et al., 2021; Tunyasuvunakool et al., 2021) (Fig. S1C), we hypothesised that this mutation would likely disrupt protein folding and induce degradation. Western blot analysis comparing Tln1 expression in *tln1^uq1al−/−^* mutants with siblings confirmed that the mutated Tln1 protein was degraded and expression was lost (Fig. 1D). Transiently knocking out Tln1 in wild-type embryos, using a published method of CRISPR/Cas9 multiplexed guide complexes (Wu et al., 2018), resulted in a phenocopy of *tln1^uq1al−/−^* mutants at 50 hpf (Fig. S1D-F). Furthermore, we did not detect evidence for genetic compensation (El-Brolosy et al., 2019) by other talin genes at the transcriptional level (Fig. S1G). Together this series of experiments validates that *tln1^uq1al^* is a null allele.

Tln1 is essential for vinculin recruitment and formation of mature FAs (Calderwood et al., 2013; Goult et al., 2018). To explore vinculin recruitment and formation of FAs in *tln1^uq1al−/−^* mutants, we generated a novel vascular-restricted vinculin transgenic line. Zebrafish have two vinculin paralogs, *vinculina* and *vinculinb* (*vcla* and *vclb*), that share equal sequence conservation with mammalian vinculin (Han et al., 2017). Both paralogs are required for the formation of junctional fingers that strengthen cell-cell junctions when exposed to flow pressure (Kotini et al., 2022). As *vinculinb* is most prominently expressed in zebrafish ECs (Lawson et al., 2020), we generated a vascular restricted transgenic line, *Tg(fli1ep:vinculinb-eGFP)^uq2al^*, whereby Vinculinb is tagged with eGFP at the C terminus. Live imaging of the main axial artery, the dorsal aorta (DA), revealed Vinculinb localisation at cell-cell junctions and at FAs in wild-type embryos (Fig. 1E and Movie 1). The presence of Vinculinb-eGFP at FAs was validated by colocalisation with another major FA protein, Paxillin (Fig. S1H). In *tln1^uq1al−/−^* mutants, Vinculinb localisation at FAs was significantly reduced, although we could still detect Vinculinb expression at cell-cell junctions (Fig. 1E-F and Movie 1). Notably, at wild-type junctions, Vinculinb was organised in a distinctive punctate pattern. In *tln1^uq1al−/−^* mutants, however, junctional clusters are significantly reduced and instead Vinculinb was distributed more continuously along the junctions (Fig. 1E,G). We did not observe a significant difference in the overall width of the junctions (Fig. 1H). These data confirm that Vinculinb-positive FAs fail to form in the absence of Tln1 and that this zebrafish model is uniquely suited to explore the consequences of FA loss on vascular morphogenesis.

### Tln1 function is required for efficient sprouting angiogenesis and network maintenance

To determine the impact of Tln1 loss on vascular morphogenesis, we first examined the vasculature at 30 h post fertilisation (hpf). To label the vasculature, we made use of our previously reported VE-cadherin transgenic line (Lagendijk et al., 2017), *TgBAC(ve-cad:ve-cadTS)^uq11bh^* (further referred to as VE-cadherin-TS). Studies using early EC-specific *Tln1* knockout mice, have identified severe defects in vasculogenesis (Monkley et al., 2011; Pulous et al., 2019). However, the main axial artery and vein, the DA and posterior cardinal vein (PCV), that form via this process developed normally in *tln1^uq1al−/−^* mutants. Tln1 protein has previously been suggested to be extremely stable and to function sufficiently for a period of time in ECs that have undergone Tln1 deletion (Monkley et al., 2011). We therefore anticipated that this difference in severity might be due to maternally deposited Tln1 protein and/or mRNA at sufficient levels to allow vasculogenesis to occur. To test this, we isolated mRNA from wild-type embryos at the one-cell stage, 1 hpf, 3 hpf, 1 dpf and 2 dpf. Indeed, we could confirm that *tln1* mRNA is maternally provided (Fig. S2A) and that this contribution is likely to be sufficient to facilitate vasculogenesis. The fact that the DA and PCV develop normally allowed us to investigate FA-deficient ECs in flow pressured vasculature following normal vasculogenesis. We therefore decided to focus on the ongoing development of the trunk vasculature for our further analysis of the *tln1^uq1al−/−^* mutants.

**Fig. 2.**
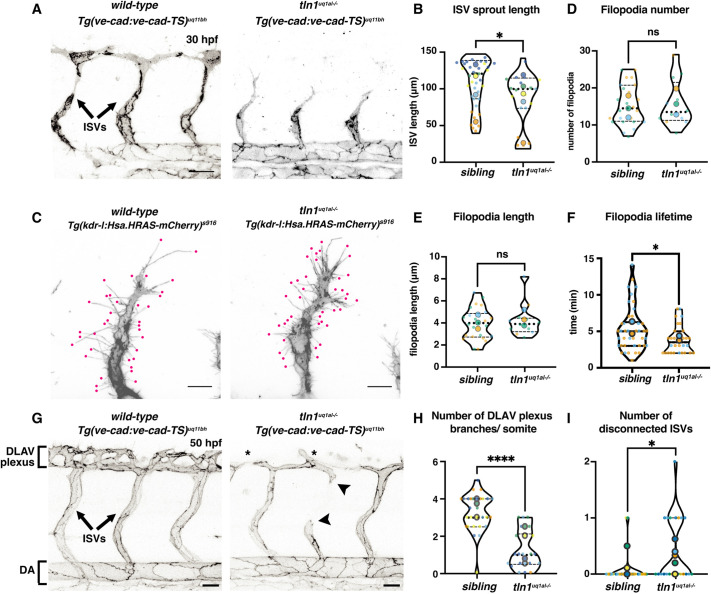
**Focal adhesion function is required for efficient angiogenic sprouting and network maintenance.** (A) Trunk vasculature of a *Tg(ve-cad:ve-cad-TS)*-positive wild-type sibling and a *tln1^uq1al−/−^* mutant embryo at 30 hpf. ISV sprouts are indicated. Scale bars: 50 µm. (B) Quantification of ISV sprout length, showing a mild reduction in mutants, *n*=5 replicates: *n*=39 ISVs in siblings and *n*=19 ISVs in *tln1^uq1al−/−^* mutants (*P<0.05, Mann–Whitney test). (C) High-resolution live image showing filopodial extensions at the sprouting front of an ISV in a wild-type sibling and in *tln1^uq1al−/−^* mutant embryo at 30 hpf. Embryos express *Tg(kdrl:Hsa.HRAS-mCherry)^s916^*, labelling the EC membrane. Scale bars: 10 µm. (D,E) Quantification of filopodia number (D) and length (E) at the tip of ISV sprouts, *n*=3 replicates: *n*=24 filopodia in siblings and *n*=12 filopodia in *tln1^uq1al−/−^* mutants, no significant difference (ns) (unpaired *t*-test) . (F) Lifetime (min) of filopodia at the sprouting tip. Lifetime is time between emergence and full retraction of a single filopodia, *n*=2 replicates: *n*=42 filopodia in siblings and *n*=30 filopodia in *tln1^uq1al−/−^* mutants (*P<0.05, Mann–Whitney test). (G) Trunk vasculature of a *Tg(ve-cad:ve-cad-TS)*-positive wild-type sibling and a *tln1^uq1al−/−^* mutant embryo at 50 hpf, showing reduced DLAV plexus formation (asterisks) and disconnected ISVs (arrowheads) in the mutant. Scale bars: 50 µm. (H) Quantification of the number of DLAV branches per somite, *n*=5 replicates: *n*=29 siblings and *n*=22 *tln1^uq1al−/−^* mutants (****P<0.0001, unpaired *t*-test). (I) Quantification of the number of disconnected ISVs across the width of two somites, located above the yolk extension, *n*=5 replicates: *n*=29 siblings and *n*=22 *tln1^uq1al−/−^* mutants (*P<0.05, Mann–Whitney test). In all graphs, replicate averages are depicted by large circles; smaller circles indicate individual data points of each replicate (colour matched).

A notable difference when imaging the vascular network at 30 hpf was the reduction in intersegmental vessel (ISV) sprout length in *tln1^uq1al−/−^* mutants (Fig. 2A,B), which could not be explained by a reduction in the number of ECs present in these sprouts (Fig. S2B,C). F-actin-rich filopodial extensions have been shown to be essential to facilitate efficient sprouting angiogenesis of ISVs in zebrafish (Phng et al., 2013). Such filopodial extensions are well known to interact and anchor to the ECM via FAs (Fischer et al., 2019). We therefore sought to determine whether altered filopodia dynamics was contributing to reduced sprouting efficiency. We first examined filopodia number and length, using a transgenic line that expresses a membranous mCherry; *Tg(kdrl:Hsa.HRAS-mCherry)^s916^*. Both the number and length of ISV filopodia at the sprouting tip were comparable between *tln1^uq1al−/−^* mutants and siblings (Fig. 2C-E). However, subsequent live monitoring of filopodia dynamics by time-lapse imaging revealed that the lifespan of filopodia was significantly reduced (Fig. 2F and Movie 2). This rapid retraction suggests that cell-ECM connectivity is compromised, contributing to reduced sprouting efficiency. Imaging at 50 hpf showed that the ISVs do eventually reach the dorsal side of the trunk and anastomose successfully to form the dorsal longitudinal anastomotic vessel (DLAV). However, the dorsally derived DLAV plexus fails to fully form (Fig. 2G,H). We further observed that the ISVs in *tln1^uq1al−/−^* mutants frequently regressed and disconnected from the main vessel network (Fig. 2G,I and Movie 3), suggesting that the network is not maintained. As *tln1^uq1al−/−^* mutants have developed mild cardiac oedema at this time point, we sought to determine whether changes in blood flow might have impacted DLAV plexus formation and network maintenance. We first quantified blood flow velocity in the DA and ISVs from high-speed live recordings and identified that although blood flow is not significantly affected in the DA, there is a reduction of flow in ISVs (Fig. S3A and Movies 4 and 5). To test whether blood flow loss could inhibit DLAV plexus formation and induces ISV vessel regression, we treated wild-type embryos with the muscle relaxant 2,3-butanedione monoxime (BDM) from 30 to 50 hpf. Treatment inhibits cardiac contraction and thus blood flow. Blood flow loss significantly compromised the formation of the DLAV plexus; however, we did not observe ISV vessel regression (Fig. S3B,C). Previous work by Sugden and colleagues also showed that stochastic loss of ISV perfusion does not lead to regression of these vessels (Sugden et al., 2017). From these data, we can conclude that reduced flow in the ISVs of *tln1^uq1al−/−^* mutants contributes to loss of DLAV plexus formation. However, FAs are likely to play an essential role in maintaining the vascular network with loss of Tln1 contributing to ISV vessel regression.

### Focal adhesions are required for endothelial cell elongation during blood vessel remodelling

**Fig. 3.**
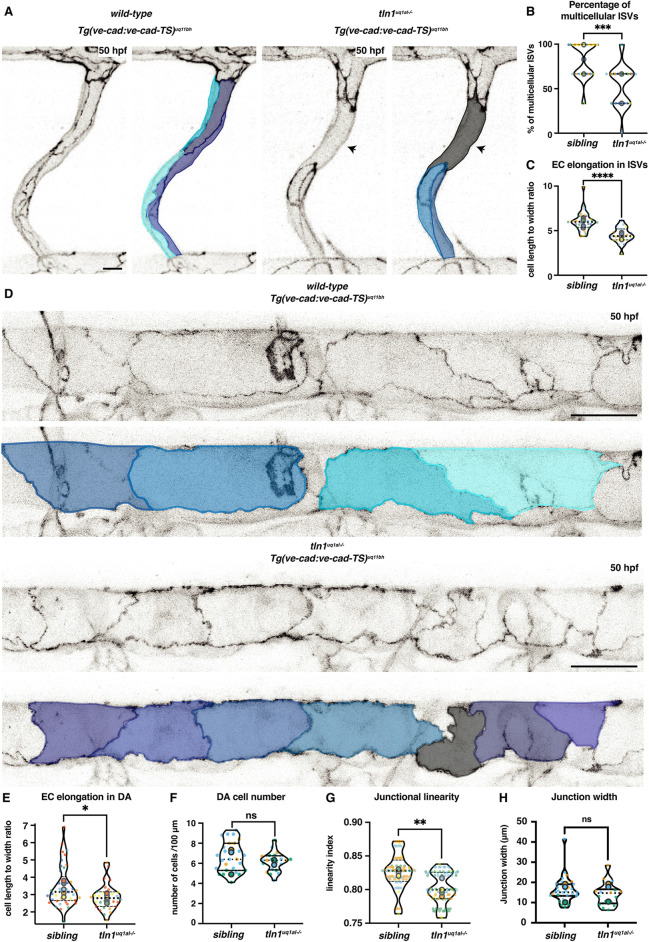
**Loss of focal adhesions impairs endothelial cell remodelling.** (A) Single ISVs of a wild-type sibling and in *tln1^uq1al−/−^* mutant embryo at 50 hpf, expressing VE-cadherin *Tg(ve-cad:ve-cad-TS)*. False-coloured images indicate EC shapes. Arrowheads indicate a region of the ISV that is unicellular in the mutant. Scale bar: 10 µm. (B) Quantification of the percentage of multicellular ISVs per two somites, *n*=5 replicates: *n*=31 siblings and *n*=22 *tln1^uq1al−/−^* mutants (***P<0.001, Mann–Whitney test). (C) Quantification of EC elongation in ISVs, *n*=5 replicates: *n*=31 siblings and *n*=22 *tln1^uq1al−/−^* mutants (****P<0.0001, Mann–Whitney test). (D) Dorsal aorta (DA) of a wild-type sibling and a *tln1^uq1al−/−^* mutant embryo at 50 hpf, expressing VE-cadherin *Tg(ve-cad:ve-cad-TS)*. False-coloured duplicate images indicate EC shapes. Scale bars: 25 µm. (E) Quantification of EC elongation in the DA, *n*=6 replicates: *n*=41 siblings and *n*=30 *tln1^uq1al−/−^* mutants (*P<0.05, Mann–Whitney test). (F) Number of ECs in the DA, quantified over a 100 µm region, *n*=3 replicates: *n*=31 sibling ECs and *n*=17 *tln1^uq1al−/−^* mutant ECs, no significant difference (ns) (Mann-Whitney test). (G) Quantification of junctional linearity of ECs in the DA, *n*=6 replicates: *n*=20 siblings and *n*=21 *tln1^uq1al−/−^* mutants (**P<0.01, unpaired *t*-test). (H) Quantification of junction width measured by VE-cadherin-TS expression, *n*=3 replicates: *n*=31 siblings and *n*=16 *tln1^uq1al−/−^* mutants, no significant difference (ns) (Mann-Whitney test). In all graphs, replicate averages are depicted by large circles; smaller circles indicate individual data points of each replicate (colour matched).

The developmental time window at which ISV regression occurs coincides with well characterised cellular remodelling events. Remodelling involves a dynamic process of EC elongation (Bentley et al., 2014; Lagendijk et al., 2017; Perryn et al., 2008; Sauteur et al., 2014; Sugden et al., 2017), which contributes to the transformation of unicellular ISVs into multicellular ISVs (Sauteur et al., 2014). In unicellular ISVs, ECs are stacked one on top of the other. Upon EC rearrangements, multicellular tubes are formed with multiple cells lining the vessel lumen, which improves vessel stability (Bentley et al., 2014; Perryn et al., 2008; Sauteur et al., 2014). To investigate whether EC remodelling might be impacted by loss of Tln1, we again made use of our VE-cadherin-TS transgenic line and performed live imaging of the trunk vasculature at 50 hpf. We found that segments of *tln1^uq1al−/−^* ISVs displayed a unicellular configuration at 50 hpf whereas ISVs in siblings progressed into a multicellular configuration (Fig. 3A,B). Quantification of cell shape revealed that the ECs that made up the *tln1^uq1al−/−^* ISVs were less elongated (Fig. 3C). Because a hallmark of multicellular tube formation is EC elongation, we suggest that the reduced capacity of *tln1^uq1al−/−^* ECs to elongate results in regions of unstable ISVs, which might contribute to ISV regression.

We and others have previously reported that ECs in the DA also undergo a process of EC elongation as this vessel matures. This process is initiated at 2 dpf (Lagendijk et al., 2017; Sugden et al., 2017) and is essential for DA vessel diameter adaptation (Sugden et al., 2017). To assess whether FAs might play a broader role in EC elongation, we examined ECs of the DA. Quantification of EC shape in *tln1^uq1al−/−^* mutant and sibling embryos at 50 hpf revealed that *tln1^uq1al−/−^* ECs were less elongated (Fig. 3D,E). Notably, the number of ECs within the DA was not changed (Fig. 3F). An additional hallmark of EC remodelling in the DA is linearisation of EC junctions, whereby irregular junctions straighten over time (Lagendijk et al., 2017). In *tln1^uq1al−/−^* mutants the ECs in the DA presented with significantly fewer linear junctions, without changes to the overall width of the junctions (Fig. 3G,H). These data are in line with previous studies in mice, where irregular VE-cadherin junctions were also identified in settings of altered cell-ECM adhesion (Pulous et al., 2019; Yamamoto et al., 2015; Zovein et al., 2010). Although we determined that the DA was still experiencing blood flow at a velocity that was comparable to siblings (Fig. S3A and Movie 4), we sought to directly test whether any mild reduction in blood flow could inhibit EC elongation. To do this, we injected of a suboptimal dose of *tnnt2a* morpholino (referred to as diluted *tnnt2a* MO) in wild-type embryos. This approach was recently established by Vignes and colleagues (Vignes et al., 2022) and we validated that injection of diluted *tnnt2a* MO reduced blood flow velocity significantly (Fig. S3D-G and Movie 6). Notably, EC elongation was not affected in the DA of diluted *tnnt2a* MO-injected embryos, and we made the interesting observation that reduced flow induced linearisation of the EC junctions. These data show that loss of EC elongation is a primary defect in *tln1^uq1al−/−^* mutants and is not caused by changes in cell proliferation or blood flow. The data further suggest that FAs regulate EC elongation in both larger calibre vessels and capillaries, and that FA-deficient ECs are characterised by irregular cell-cell junctions, which are likely to contribute to increased vessel permeability, a major phenotypic hallmark of FA loss-of-function mouse models (Lei et al., 2008; Monkley et al., 2011; Pulous et al., 2021, 2019; Tanjore et al., 2008; Yamamoto et al., 2015).

### Focal adhesions regulate endothelial cell elongation in an endothelial cell-autonomous manner

**Fig. 4.**
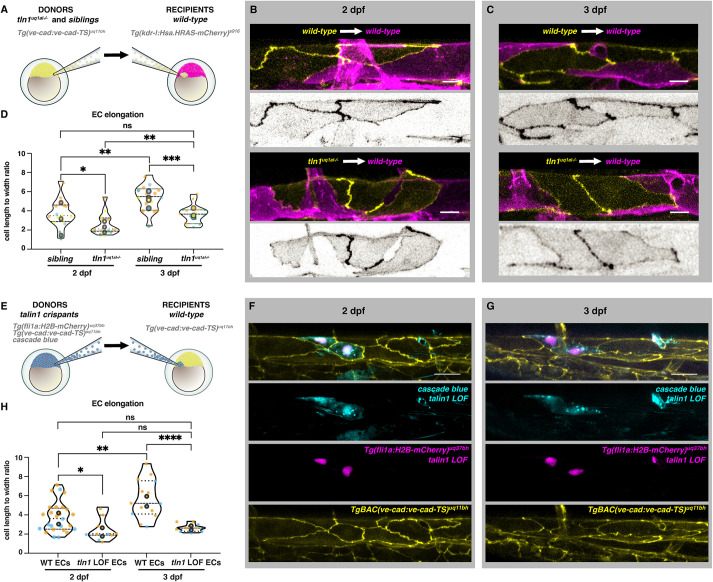
**Talin 1 is required for endothelial cell elongation cell-autonomously.** (A) Schematic representation of transplantation procedure. For these experiments, we transplanted cells at blastula stage from *Tg(ve-cad:ve-cad-TS)^uq11bh^* donors to *Tg(kdrl:Hsa.HRAS-mCherry)^s916^* recipients. (B) Wild-type dorsal aorta (magenta) of recipient embryos at 2 dpf containing either wild-type donor ECs (top, yellow) or *tln1^uq1al−/−^* mutant ECs (bottom, yellow). Scale bars: 10 µm. Greyscale images show donor ECs only. (C) Wild-type recipient dorsal aorta (magenta) of recipient embryos at 3 dpf containing wild-type donor ECs (top, yellow) or *tln1^uq1al−/−^* mutant ECs (bottom, yellow). Scale bars: 10 µm. Greyscale images show donor ECs only. (D) Quantification of EC elongation of transplanted ECs during dorsal aorta maturation from 2 dpf to 3 dpf, *n*=4 replicates; 2 dpf data, *n*=15 sibling ECs and *n*=14 *tln1^uq1al−/−^* mutant ECs; 3 dpf data, *n*=15 sibling ECs and *n*=15 *tln1^uq1al−/−^* mutant ECs. *P<0.05 for 2 dpf sibling versus *tln1^uq1al−/−^* mutant (Mann-Whitney test). ***P<0.001 for 3 dpf sibling versus 3 dpf *tln1^uq1al−/−^* mutant (unpaired *t*-test). **P<0.01 for 2 dpf sibling versus 3 dpf sibling (Mann-Whitney test). **P<0.01 for 2 dpf *tln1^uq1al−/−^* mutant versus 3 dpf *tln1^uq1al−/−^* mutant (Mann-Whitney test). (E) Schematic representation of complementary transplantation experiments. We transplanted cells from Cascade Blue-injected *tln1* crispants carrying two transgenes: *Tg(ve-cad:ve-cad-TS)^uq11bh^* and *Tg*(*fli1ep:nls-mCherry)^uq37bh^.* Cells were transplanted into *Tg(ve-cad:ve-cad-TS)^uq11bh^* wild-type embryos at blastula stages. (F) A small graft of Tln1-deficient ECs [indicated by Cascade Blue and *Tg*(*fli1ep:nls-mCherry)^uq37bh^* expression] within a wild-type DA at 2 dpf. Scale bar: 25 µm. (G) Endothelial-restricted Tln1-deficient ECs, imaged at 3 dpf. Scale bar: 25 µm. (H) Quantifications of EC shape of Tln1-deficient donor cells versus neighbouring wild-type ECs at 2 and 3 dpf, showing that Tln1-deficient cells fail to elongate while neighbouring ECs are unaffected and elongate significantly over time. Graphs show replicate averages (large circles); smaller circles indicate individual data points of each replicate (colour matched), *n*=2 replicates; 2 dpf data, *n*=25 wild-type ECs and *n*=11 *tln1^uq1al−/−^* mutant ECs; 3 dpf data, *n*=14 wild-type ECs and *n*=10 *tln1^uq1al−/−^* mutant ECs. Mann-Whitney test for all comparisons (*P<0.01, **P<0.005, ****P<0.0001).

We next aimed to identify whether the failure of ECs to elongate was due to a loss of Tln1 specifically in the ECs. We performed cellular transplantation experiments at blastula stages and transferred cells from either *tln1^uq1al−/−^* mutant or sibling donor embryos into *Tg(kdrl:Hsa.HRAS-mCherry)^s916^* wild-type recipients (Fig. 4A). We used the VE-cadherin-TS marker, which was expressed only by donor embryos, and quantified EC shape within mosaic clones in the DA. We focussed on the DA for these experiments as EC elongation in the DA is prominent from 2 dpf onwards. These quantifications confirmed that ECs require FA function to regulate their elongation during vessel remodelling (Fig. 4B-D). Ubiquitous *tln1^uq1al−/−^* mutants develop progressive cardiac failure, causing a loss of blood circulation at 3 dpf; therefore, we did not examine EC remodelling in *tln1^uq1al−/−^* mutants beyond 2 dpf. Generating chimeric embryos by transplantation, however, allowed us to examine the consequences of FA loss in ECs at later stages. Although EC elongation becomes more pronounced in ECs of wild-type origin between 2 and 3 dpf (Lagendijk et al., 2017; Sugden et al., 2017), this progressive elongation does not occur in clones of *tln1^uq1al−/−^* mutant ECs. In fact, these FA-deficient ECs at 3 dpf were indistinguishable in shape when compared with EC clones derived from sibling embryos at 2 dpf (Fig. 4D).

We next sought to directly compare Tln1-deficient ECs with neighbouring wild-type ECs. To do this, we performed similar transplantation assays. We transplanted Tln1-deficient cells from *tln1* crispants carrying two transgenes: *Tg(fli1a:H2B-mCherry)^uq37bh^*, labelling EC nuclei; and VE-cadherin-TS, labelling cell-cell junctions. Tln1-deficient cells were transplanted into VE-cadherin-TS wild-type embryos (Fig. 4E). Donor embryos were also injected with cascade blue dye, allowing us to examine grafts that were restricted to the endothelium. These experiments validated that Tln1 acts cell-autonomously as Tln1-deficient ECs were significantly less elongated compared with neighbouring wild-type ECs at both 2 dpf and 3 dpf (Fig. 4F-H).

### EC elongation requires forces generated via a functional network of F-actin fibres

Ultimately, dynamic changes in acto-myosin contractility determine cell shape. Vinculin is well known to assist in the reinforcement of FA-actin binding, allowing the enhanced force bearing (Bays and DeMali, 2017; Grashoff et al., 2010). Loss of vinculin-positive FAs (Fig. 1D,E) thus implies that *tln1^uq1al−/−^* mutant ECs are likely to be experiencing a non-physiological distribution of force. We therefore hypothesised that impairment of FAs in *tln1^uq1al−/−^* mutants disturbs actin rearrangements, which subsequently hampers the ability of ECs to elongate.

**Fig. 5.**
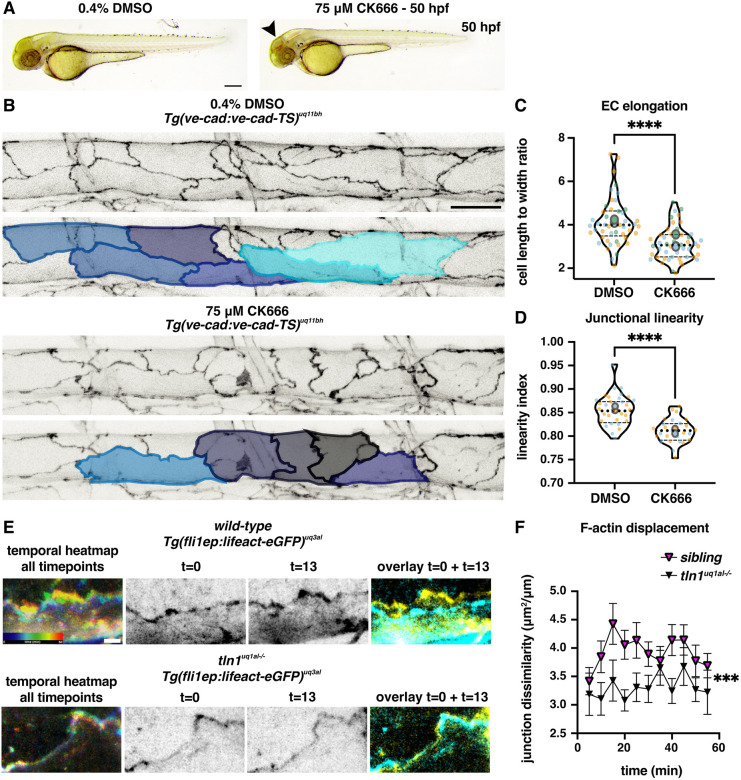
**Actin polymerisation induces replicated endothelial cell remodelling phenotypes.** (A) Bright-field images showing the overall body morphology at 50 hpf of embryos upon 5 h treatment with either 0.4% DMSO (control, left) or with 75 μM CK666 (Arp2/3 inhibitor, right). CK666-treated embryos develop cranial haemorrhaging (arrowhead), reminiscent of *tln1^uq1al−/−^* mutants (Fig. 1A). Scale bar: 100 µm. (B) Maximum projection of the dorsal aorta at 50 hpf, directly after treatment with either DMSO (control, top) or CK666 (bottom), showing impaired EC elongation. Duplicate images, one with false-coloured ECs to highlight EC shape. The colours present a spectrum: lighter colours indicate more elongated ECs. Scale bar: 25 µm. (C) Quantification of EC elongation at 50 hpf, showing a significant reduction upon CK666 treatment, *n*=3 replicates: *n*=48 DMSO- and *n*=48 CK666-treated embryos (Mann–Whitney test, ****P<0.0001). (D) Quantification of cell-cell junction linearity at 50 hpf, showing a significant reduction upon CK666 treatment, *n*=3 replicates: *n*=48 DMSO- and *n*=48 CK666-treated embryos (unpaired *t*-test, ****P<0.0001). (E) Still images from time-lapse imaging (Movie 8). Movie records cortical F-actin rearrangements in wild-type (top) and *tln1^uq1al−/−^* mutant ECs (bottom) from 48 to 49 hpf. (Left) Temporal colour-coded projection of 13 timepoints imaged during the 1 h time-lapse. (Middle) Greyscale images of F-actin expression at the start (t=0) and the end of the movie (t=13). (Right) Overlay of *t*=0 (cyan) and *t*=13 (yellow) showing F-actin displacement in the wild type and very little movement in *tln1^uq1al−/−^* mutant. Scale bar: 5 μm. (F) Quantification of F-actin displacements of cortical F-actin over the course of a 1-h time-lapse movie in siblings (magenta) and *tln1^uq1al−/−^* mutants (black) (Mann-Whitney test, ***P<0.001). Each data point represents the average displacement between two consecutive time-points, *n*=3 replicates: *n*=20 siblings and *n*=14 *tln1^uq1al−/−^* mutants. In C and D, replicate averages are depicted by large circles; smaller circles indicate individual data points of each replicate (colour matched).

To test this hypothesis, we first examined whether forces generated by polymerised F-actin fibres are required for EC elongation during DA maturation. To do this, we used a well-characterised chemical inhibitor of the Arp2/3 complex, CK666 (Cao et al., 2017; Phng et al., 2015). We treated wild-type VE-cadherin-TS embryos with CK666 (75 μM) from 24 hpf to 50 hpf, and subsequently imaged the DA. Similar to *tln1^uq1al−/−^* mutants, CK666-treated embryos presented with bleedings in the brain (Fig. 5A), a smaller DLAV plexus (Fig. S4A,B) and multicellular ISV tube formation (Fig. S4A,C). Upon CK666 treatment, blood flow velocity in the DA was reduced at a level comparable with that of diluted *tnnt2* MO-injected embryos (Fig. S4D and Movie 7). However, although flow reduction alone did not impact EC elongation (Fig. S3D,E), CK666 treatment strongly inhibited EC elongation (Fig. 5B,C) and the ECs were bounded by irregular cell-cell junctions (Fig. 5D). Shorter treatment during angiogenesis also resulted in stunted ISV formation (Fig. S4E,F). The similarity in EC remodelling phenotypes between CK666-treated embryos and *tln1^uq1al−/−^* mutants suggested that when FA function is compromised, F-actin organisation and movement are perturbed.

We then used a vascular F-actin marker line, *Tg(fli1ep:lifeact-eGFP)^uq3al^*, to examine F-actin rearrangements in *tln1^uq1al−/−^* animals*.* As we have observed changes in cell-cell junction linearisation simultaneously with EC elongation, we hypothesise that FAs drive actin rearrangements that can act on cortical actin fibres near cell-cell junctions. Paatero and colleagues have previously identified junction-based lamellipodia (JBLs) at cell-cell junctions (Paatero et al., 2018). F-actin protrusions in JBLs bring in junctional proteins, thereby remodelling junction morphology. To generate movies at high temporal resolution, we imaged cortical actin at 5 min intervals for 1 h starting at 48 hpf (Fig. 5E and Movie 8). In *tln1^uq1al−/−^* mutants, the overall displacement of F-actin over the 1 h time-lapse was significantly reduced (Fig. 5F), reflecting the failure of cells to elongate. This analysis demonstrated a reduction in F-actin rearrangements in FA-deficient ECs, compared with ECs of sibling embryos. These observations suggest that, in the absence of FA function, F-actin rearrangements are altered, preventing EC elongation and cell-cell junction elongation.

### Promoting polymerised F-actin networks can compensate for Focal adhesions loss

**Fig. 6.**
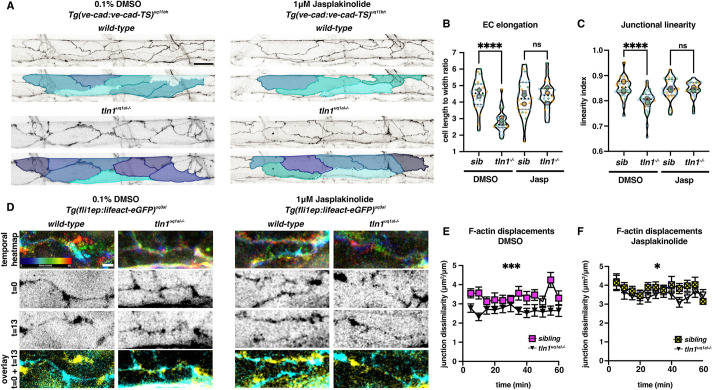
**Enhancing polymerised F-actin can restore EC elongation, junctional linearity and F-actin rearrangements in *tln1^uq1al−/−^* mutants.** (A) Maximum projection of the dorsal aorta at 50 hpf, directly after treatment with either 0.1% DMSO (control, left) or 1 μM jasplakinolide (Jasp, right) showing impaired EC elongation in DMSO-treated *tln1^uq1al−/−^* mutants (bottom left), which is rescued when mutants have been exposed to Jasp (bottom right). The colours present a spectrum: lighter colours indicate more elongated ECs. Scale bar: 25 µm. (B) Quantification of EC elongation at 50 hpf, showing the phenotypic rescue of EC elongation in Jasp-treated *tln1^uq1al−/−^* mutants (*n*=3 replicates): *n*=27 siblings and *n*=25 *tln1^uq1al−/−^* mutants in DMSO-treated group; *n*=27 siblings and *n*=22 *tln1^uq1al−/−^* mutants in Jasp-treated group. ****P<0.0001 for DMSO sibling versus *tln1^uq1al−/−^* mutant (Mann-Whitney test). For Jasp sibling versus *tln1^uq1al−/−^* mutant, no significant difference (ns) (*t*-test). (C) Quantification of junctional linearity at 50 hpf, showing significant rescue of linearisation in Jasp-treated *tln1^uq1al−/−^* mutants, *n*=3 replicates: *n*=27 siblings and *n*=33 *tln1^uq1al−/−^* mutants in DMSO-treated group; *n*=28 siblings and *n*=25 *tln1^uq1al−/−^* mutants in Jasp-treated group. ****P<0.0001 for DMSO sibling versus *tln1^uq1al−/−^* mutant (Mann-Whitney test). For Jasp sibling versus *tln1^uq1al−/−^* mutant, no significant difference (ns) (Mann–Whitney test). (D) Stills from time-lapse imaging (Movies 9 and 10) of cortical F-actin displacements in 0.1% DMSO (control, left) or 1 μM Jasp-treated (right) wild-type siblings and *tln1^uq1al−/−^* mutant embryos. Each column of images is organised as follows: top, temporal colour-coded projection of 13 timepoints imaged during the hour time-lapse; middle, greyscale images of F-actin expression at the start (t=0) and the end of the movie (t=13); bottom, overlay of *t*=0 (cyan) and *t*=13 (yellow) showing F-actin displacement. Scale bar: 5 μm. (E) Quantification of cortical F-actin displacement over the course of a 1 h time-lapse movie in 0.1% DMSO-treated siblings (yellow) and *tln1^uq1al−/−^* mutants (black). Scale bar: 10 µm. ***P<0.001 (Mann-Whitney test). (F) Quantification of cortical F-actin displacements over the course of a 1 h time-lapse movie in Jasp-treated siblings (yellow) and *tln1^uq1al−/−^* mutants (black) showing a partial rescue when compared with E, *n*=3 replicates: *n*=12 siblings and *n*=12 *tln1^uq1al−/−^* mutants in DMSO-treated group; *n*=14 siblings and *n*=10 *tln1^uq1al−/−^* mutants in Jasp-treated group (*P<0.05, Mann–Whitney test). In B and C, replicate averages are depicted by large circles; smaller circles indicate individual data points of each replicate (colour matched).

We next aimed to determine whether stabilising and enhancing polymerised F-actin in *tln1^uq1al−/−^* mutants was sufficient for EC elongation. To do this, we used jasplakinolide (Jasp), a compound that stabilises actin filaments by reducing disassembly and promotes polymerisation through F-actin nucleation (Bubb et al., 2000; Holzinger, 2009). We treated *tln1^uq1al−/−^* mutant and sibling embryos with either Jasp (1 μM) or 0.1% DMSO from 24 hpf until 50 hpf, when the embryos were imaged. We observed an improvement in DLAV plexus formation (Fig. S4G,H), and ISVs more frequently matured into multicellular tubes in Jasp treated *tln1^uq1al−/−^* mutants (Fig. S4G,I), indicating that DLAV sprouting and EC remodelling can be partially restored to FA-deficient ECs by promoting polymerisation of F-actin. We further quantified cell shape of ECs in the DA, which revealed that EC elongation and junction linearisation were rescued in Jasp-treated *tln1^uq1al−/−^* mutants (Fig. 6A-C).

We next examined whether the rescue of EC elongation was reflected by improved F-actin rearrangements. We treated *tln1^uq1al−/−^* mutants and siblings with Jasp for 5 h before time-lapse imaging. As previously observed, junctional F-actin was static in 0.1% DMSO-treated *tln1^uq1al−/−^* embryos (Fig. 6D,E and Movie 8); however, F-actin displacements were more pronounced in Jasp-treated *tln1^uq1al−/−^* embryos (Fig. 6D,F; Movies 9 and 10). Taken together, these experiments show that enhancing F-actin polymerisation and promoting the stability of these networks can result in sufficient acto-myosin activity for elongation and junction linearisation of FA-deficient ECs. Whether compensation occurs via the few remaining FAs (Fig. 1E) or whether elongation is mainly driven by JBLs at cell-cell junctions remains to be determined.

## DISCUSSION

This study reports the first live *in vivo* analysis of FA-deficient endothelial cells in angiogenesis and vascular remodelling during development. We generated a novel *talin1* zebrafish mutant model, *tln1^uq1al^*, wherein an in-frame mutation in the F1 FERM domain results in degradation of Tln1 protein. Using a novel endothelial restricted Vinculinb-eGFP transgenic line, we were able to verify a loss of FAs in these *tln1^uq1al−/−^* mutants. Live imaging of Vinculinb further revealed dynamic behaviour of FAs in wild-type ECs. To our knowledge, this Vinculinb-eGFP transgenic line represents the first *in vivo* model that reports FA dynamics in ECs of live vasculature and therefore will provide a unique resource to the cardiovascular research community for future studies into FA function.

We uncovered that FA function is required for EC elongation and cell-cell junction linearisation in both angiogenic ISVs and in the main axial artery: the DA. This reduced capacity of ECs to elongate resulted in a failure of the vascular network to remodel and mature. The ISVs frequently presented as unicellular tubes. We hypothesise that such unicellular regions would be more fragile once exposed to blood flow, owing to high pressure inflicted onto a single EC, instead of being distributed over multiple cells. In support of this, we found that a significant proportion of the ISVs detached from the main vascular network at 2 dpf. A further explanation for the failure of ECs to reorganise appropriately in ISVs might be through a weakening of cell-cell junctions via FA-cadherin crosstalk. VE-cadherin at cell-cell junctions has previously been shown to be essential for EC elongation during ISV multicellular tube formation (Paatero et al., 2018; Phng and Belting, 2021; Sauteur et al., 2014). This failure of ISVs to remodel can explain the severe systemic haemorrhaging that has been observed in mouse models where Tln1 was depleted specifically during the developmental time window of angiogenesis (Monkley et al., 2011).

We further showed that Tln1 was also necessary for EC elongation and cell-cell junction linearisation of larger calibre vessels, specifically in the DA, and that this role was endothelial cell-autonomous. Previous studies have demonstrated that EC elongation is an essential process that occurs during DA maturation (Lagendijk et al., 2017; Sugden et al., 2017). When elongation is impaired, the DA will fail to narrow over time, which leads to systemic perfusion defects (Sugden et al., 2017). Concurrently, the EC junctions also continued to display an immature irregular morphology, instead of linearising from 2 to 3 dpf (Lagendijk et al., 2017; Sugden et al., 2017). This irregular VE-cadherin patterning is in agreement with previous observations made from EC-specific integrin-β1 (*Itgb1*) and *Tln1* knockout mice (Pulous et al., 2021; Yamamoto et al., 2015), where VE-cadherin was found to be distributed over a wider area at the cell-cell junctions. By using small-molecule inhibitors that alter actin polymerisation, we showed that inhibition of F-actin polymerisation phenocopied *tln1^uq1al−/−^* mutants*,* with a significant reduction in EC elongation and junction linearisation. Furthermore, enhancement of actin polymerisation could restore EC elongation and junction linearisation in *tln1^uq1al−/−^* mutants. Together with our observation that Tln1 deficiency results in a loss of Vinculinb clusters both at FAs and cell-cell junctions, we suggest that significant FA-cadherin crosstalk exists via acto-myosin interactions to control EC elongation and cell-cell junction linearisation.

Precisely where and when acto-myosin acts to allow EC elongation during vascular remodelling remains to be determined. Notably, these cellular processes might also be dependent on Tln1 function at cell-cell junctions, given that Pulous and colleagues have shown previously that Talins are expressed at cell-cell junctions in cultured human umbilical cord venous ECs (HUVECs) and human dermal microvascular endothelial cells (HDMVECs) (Pulous et al., 2021). Whether Tln1 is also localised at endothelial cell-cell junctions *in vivo*, however, remains to be determined. Phenotypic rescue of EC elongation in *tln1^uq1al−/−^* by Jasplakinolide, a compound known to stabilise and enhance F-actin polymerisation, also supports the idea that redundant FAs might exist, linked to actin via other adaptor proteins such as kindlins.

Overall, our findings reveal a previously unappreciated role for Tln1, and more broadly for FAs, in EC elongation and junction linearisation. The failure of these process resulted in unstable vasculature with irregular cell-cell junctions, explaining the occurrence of haemorrhaging vasculature across a range of FA-deficient animal models. As the control of vascular adhesion is essential for the maintenance of a functional vasculature and for life, it seems likely that FAs and talin proteins play a central role.

## MATERIALS AND METHODS

### Zebrafish stocks and husbandry

All animal work adhered to the guidelines of the animal ethics committee at the University of Queensland. Published transgenic lines used for this work are *TgBAC(ve-cad:ve-cadTS)^uq11bh^* (Lagendijk et al., 2017) and *Tg(kdrl:Hsa.HRAS-mCherry)^s916^* (Hogan et al., 2009). For live imaging purposes, all embryos were treated with 0.0003% phenylthiourea (PTU) from 24 hpf to prevent pigmentation and anaesthetised in Tricaine (Sigma-Aldrich E10521-50G, 0.08 mg/ml).

### Cloning and transgenesis

To establish *Tg(fli1ep:vinculinb-eGFP)^uq2al^* we amplified vinculinb cDNA and cloned it into the Gateway pME vector (pDON-221) using Gateway technology (Hartley et al., 2000). Oligos used to amplify vinculinb were as follows: vinculinb-forward, 5′-**GGGGACAAGTTTGTACAAAAAAGCAGGCT**ACCATGCCGGTTTTCCACACG-3′ (bold indicates gateway homology arm, underline indicates the Kozac sequence); vinculinb-reverse: 5′-**GGGGACCACTTTGTACAAGAAAGCTGGGTA**CTGGTACCAGGGTGTCTTGC-3′ (bold indicates gateway homology arm). Subsequently a Gateway LR reaction was performed combining a p5E-fli1ep, pME-Vinculinb and p3E-eGFP, placing the final *fli1ep:vinculinb-eGFP* sequence into pDestTol2pA2AC (containing the α-crystallin promoter driving GFP in the zebrafish lens). *Tg*(*fli1ep:lifeact-eGFP)^uq3al^* was generated by injection of a published plasmid (Phng et al., 2013) [a gift from Holger Gerhardt (Max-Delbrück-Centrum für Molekulare Medizin, Berlin, Germany) and Li-Kun Phng (RIKEN Center for Biosystems Dynamics Research, Kobe, Japan)]. For transgenesis, we injected 1 nl of purified plasmid DNA (100 ng/μl) into single-cell stage embryos together with tol2 transposase mRNA (25 ng/μl). Embryos expressing Vinculinb-eGFP or Lifeact-eGFP were selected mosaically and raised to adulthood. Adults were screened for germline transmission to generate stable transgenic lines. Plasmids, *Tg(fli1ep:vinculinb-eGFP)^uq2al^* transgenics and *Tg*(*fli1ep:lifeact-eGFP)^uq3al^* transgenics are available from the corresponding author on request.

### Western blot analysis

3 dpf *tln1^uq1al−/−^* mutant and sibling embryos were collected into pre-chilled 1.5 ml Eppendorf tubes. Embryos were homogenised in sample buffer [one embryo/5µl of samples buffer: 50 mM Tris-HCl (pH 6.8); 2% SDS; 1.5 mM bromophenol blue; 10% glycerol; 100 mM DTT; 1× protease inhibitor (cOmplete, Merck, 5056489001)] using Bel-Art disposable polypropylene pestles (Merck, BAF199230001). Homogenised samples were heated at 95°C for 10 min. Subsequently, 40µl of each sample was resolved on 8-12% polyacrylamide gels, transferred to nitrocellulose membranes, followed by blocking with 5% milk in TBS-T. The blots were washed three times with TBS-T and incubated with mouse-anti-Talin (C9) (Santa Cruz Biotechnology, sc-365875, 1:200 dilution) and rabbit-anti-GAPDH (Abcam, AB181603, 1:10,000 dilution) antibodies in 3% BSA overnight at 4°C. This was followed by three washes with TBS-T and incubation with secondary antibodies for1 h at room temperature [Bio-Rad Laboratories, goat-anti-mouse IgG (H+L)-HRP conjugate 1706516 and goat-anti-rabbit IgG (H+L)-HRP conjugate 1706515, both diluted 1:5000 in 5% milk powder in TBS-T].

### Genome editing and genotyping

To generate the *tln1^uq1al^* line, CRISPR genome editing for *talin1* was performed as previously described (Gagnon et al., 2014). Oligo sequence used for guide RNA transcription: 5′-*taatacgactcactata*GGGCGGACGCTGCGGGAGCGTTTTAGAGCTAGAAATAGCAAG-3′ (italics indicate the T7 site for transcription; underline indicates guide RNA sequence).

To generate a transient knockout model for *talin1* (referred to as *talin1* crispants), we used a published approach of multiplexed guide injections (Wu et al., 2018). Sequences of the four guides used to target *talin1* simultaneously were as follows: guide 1, 5′-*taatacgactcactata*GGATCATGGGCGGACGCTGCGTTTTAGAGCTAGAAATAGC-3′; guide 2, 5′-*taatacgactcactata*GGCAGACGCCTGGTCACTGAGTTTTAGAGCTAGAAATAGC-3′; guide 3, 5′-*taatacgactcactata*GGTCAGCCAGAGCACTGGCCGTTTTAGAGCTAGAAATAGC-3′; guide 4, 5′-*taatacgactcactata*GGCAGGCAATCGTGGCACTCGTTTTAGAGCTAGAAATAGC-3′.

To genotype the *tln1^uq1al−/−^* allele, we amplified a 124 bp region with primers flanking the mutation site. Amplified products were separated using 2% sodium borate agarose gels. Genotypes were distinguished as follows: heterozygotes, two bands of 124 bp and 107 bp; homozygous wild type, single band of 124 bp; and homozygous mutant, single band of 107 bp. Primer sequences for genotyping PCR: Talin1-forward, 5’-CGCTGATTGGGATTTTAATGTGTATTCAG-3′, Talin1-reverse, 5′-CTGGTCTGAGTAGAAGAACTTTCTCC-3′.

### Immunofluorescence

To positively identify focal adhesions in the endothelium, we injected 4% PFA into the blood stream of *Tg(fli1ep:vinculinb-eGFP)^uq2al^* anaesthetised wild types at 50 hpf. Once cardiac contraction had stopped, the embryos were fixed whole mount in 4% PFA overnight and staining was performed as previously described (Lagendijk et al., 2011). Briefly, after fixing, embryos were washed in PBS-T (0.3% Triton-X100/PBS) three times, incubated in proteinase K for 10 min and washed again three times with PBS-T. Subsequently, embryos were incubated in blocking buffer (10% goat serum in PBS-T) for 1 h and with primary antibodies in blocking buffer at 4°C overnight. Washing and blocking steps were repeated the next day and embryos were incubated with secondary antibodies in blocking buffer at 4°C overnight. Secondary antibodies were removed the next day followed by three subsequent washes with PBS-T. Embryos were stored in PBS-T up to 1 week for imaging. Antibodies used were as follows: rabbit-anti-phospho-paxillin(Y118) (Thermo Fisher Scientific, 44-722G0, 1:200 dilution), chicken-anti-GFP (Abcam, ab13970, 1:200 dilution), Alexa 647 goat-anti-rabbit (Thermo Fisher Scientific, A32733, 1:500 dilution) and Alexa 488 goat anti-chicken (Thermo Fisher Scientific, A11039, 1:500 dilution).

### Reverse transcription polymerase chain reaction

For quantitative RT-PCRs (Fig. S1G), RNA was extracted from whole embryo lysates at 2 dpf using the Directzol RNA mini kit (Zymo). cDNA was synthesised and amplified using Thermo Fisher SuperScript II Reverse Transcriptase Kit. qPCR was performed on an Applied Biosystems Viia 7 384-well real-time PCR machine. Fold changes were calculated relative to housekeeping genes *rpl13* and *hprt*.

For RT-PCRs during maternal-zygotic transition (Fig. S2A), RNA was extracted from 20 wild-type embryos (pooled) at the one-cell stage, 1 hpf, 3 hpf, 1 dpf and 2 dpf. cDNA was synthesised and amplified using Thermo Fisher SuperScript II Reverse Transcriptase Kit. We included previously verified control genes (Harvey et al., 2013), including; *cyclinb1* (maternally provided), C18H16orf7 (maternally provided, but not transcribed after zygotic transition) and *tal1* (not maternally provided). Primers sequences were as follows: *tln1* fwd, 5′-GGTCTCATTTCAGCTGCTCG-3′; *tln1* rev, 5′-CGCCCTCTTGACAGCATTAC-3′; *cyclinb1* (control gene maternally provided) fwd, 5′-TCCATGTTCCTCCGTCTCTC-3′; *cyclinb1* rev, 5′-CATGTGCATCTGCTTCTGGT-3′; C18H16orf7 (maternally provided, but not transcribed after zygotic transition) fwd, 5′-TGTCCCATCTCTCCACATCA-3′; C18H16orf7 rev, 5′-GTGAGAAGGAACCCAGTCCA-3′; *tal1* (not maternally provided) fwd, 5′-ATGGCTCCATGCACACACTA-3′; *tal1* rev, 5′- GTTTCCTTGGCAACACCATT-3′.

### Embryonic transplantation

Transplantation was performed as previously described (Hogan et al., 2009). For Fig. 4A-C, donor embryos were derived from *tln1^uq1al+/−^* inter crosses [with transgenic background *TgBAC(ve-cad:ve-cadTS)^uq11bh^*]. Cells were transferred at blastula stages into *Tg(kdrl:Hsa.HRAS-mCherry)^s916^* wild-type hosts. Donor embryos were genotyped for the *tln1^uq1al^* allele immediately after transplantation.

For Fig. 4D-F, donor embryos were injected with Cascade Blue (Life Technologies, 10,000 MW) and *talin1* guide cocktail at the one-cell-stage. Donors were double transgenic for *TgBAC(ve-cad:ve-cadTS)^uq11bh^* and *Tg*(*fli1ep:nls-mCherry)^uq37bh^*. Cells from donor were transplanted into *TgBAC(ve-cad:ve-cadTS)^uq11bh^* wild-type recipients at blastula stages. Cellular transfer was performed using CellTram 4R Oil hydrolic microinjector (Eppendorf).

### Chemical treatments

For CK666 (Arp2/3 inhibitor) treatments, embryos were incubated in E3 medium containing 75 μM CK666 in 0.4% DMSO or 0.4% DMSO only as a negative control. For jasplakinolide (Jasp), embryos were incubated in E3 medium containing 1μM Jasp in 0.1% DMSO or 0.1% DMSO in E3 as a negative control. For 2,3-butanedione monoxime (BDM) treatment, embryos were incubated in E3 medium containing 25 mM BDM or in E3 medium only.

### Image acquisition

Embryos were mounted in 35 mm glass bottom dishes using 0.7% low melting point agarose. Confocal *z*-stacks were acquired on a LSM 710 Meta BiG (Zeiss) and a LSM 880 FastAiry (Zeiss) using a LD C-Apochromat W/40×1.1 NA objective. Timelapse imaging was performed on a Dragonfly Spinning Disc microscope (Andor) using Apo λ LWD W/40×1.15 NA. Bright-field images of the whole-mount zebrafish embryos were taken on a Nikon SMZ1270i with Tucsen Michrome 6 camera. Movies of blood flow (Movies 4-7) were recorded with a Prime BSI Express camera at 100 frames per second and captured using a EC-Plan Neofluar 20×0.8 NA objective on an Axio Observer 7 inverted microscope (Zeiss).

### Image processing and quantifications

All image processing and analysis were performed by using the Fiji, ImageJ (National Institutes of Health) (Schindelin et al., 2012). To ensure consistency, all quantifications of ECs in the ISVs and the DA were performed in a specific section of the trunk, above the yolk extension.

#### Vinculinb-positive clusters

A 10 pixel radius was used as a median filter and applied to the images. The images were thresholded to select for Vinculinb expression. ‘Analyse particles’ (Fiji, ImageJ) was applied to create a masks for junctional and cytoplasmic Vinculinb-clusters (cytoplasmic clusters, <5 μm^2^; junctional clusters, >5 μm^2^). The masks were applied to the original images and the ‘Find maxima’ function (prominence >15) was used to count the number of clusters in each area.

#### Cell-cell junction width

For Vinculinb expression (Fig. 1H), a 10 pixel radius was used as a median filter and applied to the images. The images were thresholded to select for Vinculinb expression. ‘Analyse particles’ was performed on the thresholded images to create a mask for junctional area based on particle size (>5 μm^2^). The original image was masked and thresholded to select for Vinculinb expression. The threshold area was measured as the junction area. The threshold images were then skeletonised, and the length of the skeleton was measured as the junction length. The junction area was divided by the junction length to yield the junction width.

For VE-cadherin junction width (Fig. 3H), a region of interest (ROI) was drawn around a cell-cell junction. The ROI was thresholded and measured for junction area. The threshold image was then skeletonised and measured for junction length. Junction width was calculated by dividing junction area by junction length.

#### Filopodia number and length

The number of filopodia within the first 10μm of the ISV tip was counted manually. The length of filopodia within the first 10μm of the ISV sprout was measured using the freehand line tool (Fiji, ImageJ).

#### Filopodia lifetime

Filopodia within 5μm of the tip of ISV sprout were identified. We quantified filopodia that emerged and retracted during the 15 min timelapse. The time between emergence and complete retraction of these filopodia was noted as the filopodia lifetime.

#### Blood flow velocity

For each blood flow recording, three red blood cells were tracked from the start of ventricular systole until it left the field of view. The blood flow velocity was calculated by dividing distance travelled by the time taken to leave the field of view.

#### Number of DLAV plexus branches

The number of dorsal vessel branches at the DLAV plexus within the last two somites above the yolk extension was quantified manually.

#### Number of disconnected ISVs

Number of disconnected ISVs within the last three somites above the yolk extension (Fig. 2I) or within 6 body segments (Fig. S3C) was counted manually in each embryo.

#### ISV sprout length

ISV sprout length was measured by using the line tool (Fiji, ImageJ) and drawing a line from the base of the sprout at the level of the DA to the tip of the sprout.

#### ISV EC number

The total number of EC nuclei within the last three ISVs above yolk extension was quantified and averaged to result in an average number of nuclei per ISV.

#### DA EC number

The total number of DA nuclei within the last two somites above yolk extension was counted. The number of nuclei was normalised by the length of DA quantified.

#### Percentage of multicellular ISV

We employed previously described methodology to distinguish unicellular from multicellular sections of the ISVs (Sauteur et al., 2014). The percentage was calculated by the number of multicellular ISVs out of the three ISVs captured in each image.

#### Endothelial cell elongation

Using the freehand tool (Fiji, ImageJ), a region of interest (ROI) was drawn to outline individual ECs [based on *TgBAC(ve-cad:ve-cadTS)^uq11bh^* expression]. We subsequently measured cell elongation by using the ‘fit ellipse’ shape measurement function (Fiji, ImageJ). Cellular ellipticity was calculated by dividing the ellipse length by the ellipse width.

#### Junctional linearity

The junctional linearity index was calculated by dividing the distance between junctional vertices over the length of the junction (Lagendijk et al., 2017). The closer to 1 in the index, the straighter the junction.

#### F-actin displacement

We measured the area by which F-actin expression had shifted between two consecutive time-points of the movies. To do this, we generated an overlay of *Tg*(*fli1ep:lifeact-eGFP)^uq3al^* expression and measured the surface area between the two. We subsequently normalised this by the average junction length of the two time points.

### Statistical analysis

The number of experiments to perform was based on our animal ethical requirements and on analogy with previous work with zebrafish; we have presented all measurements that were taken. Sample sizes are comparable with those reported for analysis of junctional dynamics in zebrafish (Lagendijk et al., 2017; Sauteur et al., 2014; Sugden et al., 2017). For each experimental comparison, embryos were randomly distributed, thus all data are acquired from sibling-matched controls. For analysis of *tln1^uq1al^* mutants at 2 dpf, we excluded occasional embryos wherein flow pressure in the DA was lost to ensure all ECs were analysed as part of a perfused vasculature network. Image acquisition and quantification of data from *tln1^uq1al^* mutants and siblings preceded genotyping; hence, analysis was genotype blinded. We performed all statistical analysis using Prism 9 (GraphPad). Prism Violin plots were combined with ‘all dot plots’ using the web-based software SuperPlotsOfData (Lord et al., 2020), providing a transparent display and quantitative comparison between experimental replicates. A D'Agostino-Pearson test was applied to test normal distribution of the data points. When the data were normally distributed, an unpaired Student's *t*-test was used for comparison of two means. When the data did not follow a normal distribution, a Mann–Whitney test was used for comparison of two means. The threshold for significance was taken as *P*<0.05 and all the data are represented as mean±s.e.m. For each experimental comparison, embryos were randomly distributed and sibling matched.

## Supplementary Material

10.1242/develop.200454_sup1 Supplementary information
