# Supplementary material for: Dynamically regulated focal adhesions coordinate endothelial cell remodelling in developing vasculature
Source: Development. 2022 Dec 12;149(23):dev200454. doi: 10.1242/dev.200454 (PMC10214536; doi:10.1242/dev.200454)
Supplement: Supplementary information [file develop-149-200454-s1.pdf]

Supplementary Figure 1

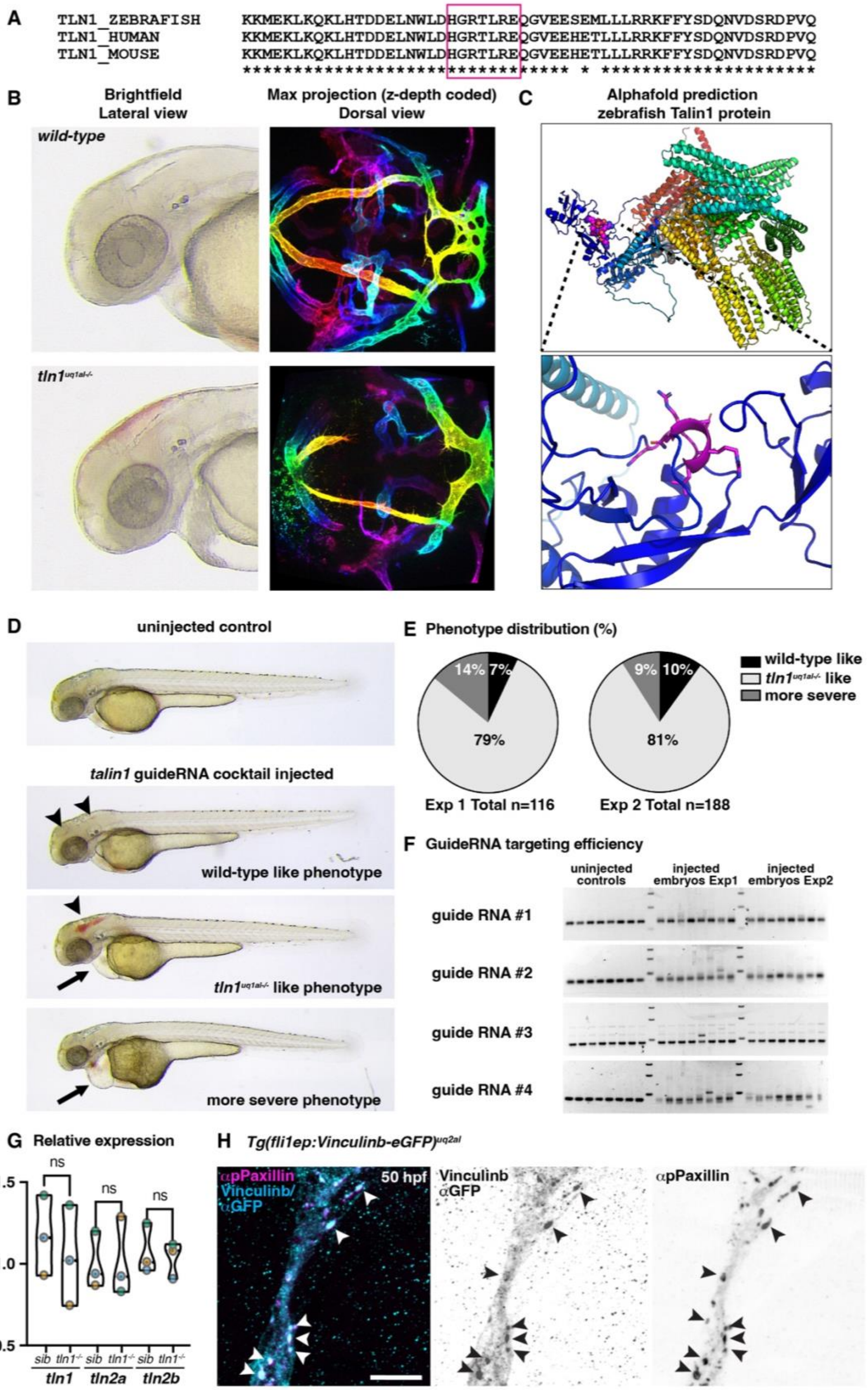

Fig. S1. Characterisation of *tln1*$^{uq1al-/-}$ allele, *talin1 crispants* and *Tg(fli1ep:Vinculinb-eGFP)*^{uq2al}

(A) Multi-species protein alignment of the CRISPR-Cas9 targeted region within the highly conserved F1 FERM domain of Talin1 in zebrafish (top), human (middle) and mouse (bottom). The box indicates the amino acids that have been deleted in our *tln1*$^{uq1al-/-}$ mutant line. (B) Left: Brightfield images of *tln1*$^{uq1al-/-}$ mutant and sibling at 48 hpf, showing cranial haemorrhaging in the mutant. Right: Max project of live confocal imaging of the cranial vasculature of these embryos, showing a loss of complexity in the muatnt. Projection is colour coded for z-dept. (C) Top: Schematic model showing the predicted structure of zebrafish Talin1 based on AlphaFold entry A0A0R4IDZ8. The model is coloured in a spectrum from blue to red from N- to C-terminus. Blue presents the N-terminal FERM domain and in magenta are the amino acids deleted in *tln1*$^{uq1al-/-}$ mutants. Bottom: Detailed view showing the wild-type structure and orientation of amino acids (magenta) that are not present in our *tln1*$^{uq1al-/-}$ mutants. (D) Brightfield images of un-injected control embryo and *talin1 crispants* at 50 hpf. Phenotypes of *talin1 crispants* are categorised into wild-type like (focal bleedings, arrowheads), *tln1*$^{uq1al-/-}$ phenocopy (prominent bleedings and mild cardiac edema, arrowhead and arrow respectively), and a "more severe phenotype" when compared to *tln1*$^{uq1al-/-}$ (prominent cardiac defects and no circulation). (E) Distribution of *talin1 crispant* phenotypes from two independent experiments (>n=100 injected embryos per experiment), showing that ~80% of the injected embryos are phenocopies of *tln1*$^{uq1al-/-}$ mutants. (F) PCR analysis of the amplicon targeted by each of the four guides in the injected guideRNA cocktail (8 injected embryos form each of the two independent experiments), showing a loss of distinctive bands and the appearance of additional amplicons of different sizes in injected embryos. (G) Quantitative RT-PCR analysis of *tln1*, *tln2a* and *tln2b* expression levels in sibling and *tln1*$^{uq1al-/-}$ mutant embryos at 2 dpf, n=3 biological replicates, n=30 siblings and n=30 *tln1*$^{uq1al-/-}$ mutants. Unpaired t-tests for all analysis revealed no significant (ns) difference for all transcripts. Replicate averages are depicted by the circles. (H) Immunofluorescence analysis revealing co-localisation (arrowheads) of Vinculinb-eGFP with p-Paxillin at FAs of ISVs at 50 hpf (n=10 embryos).

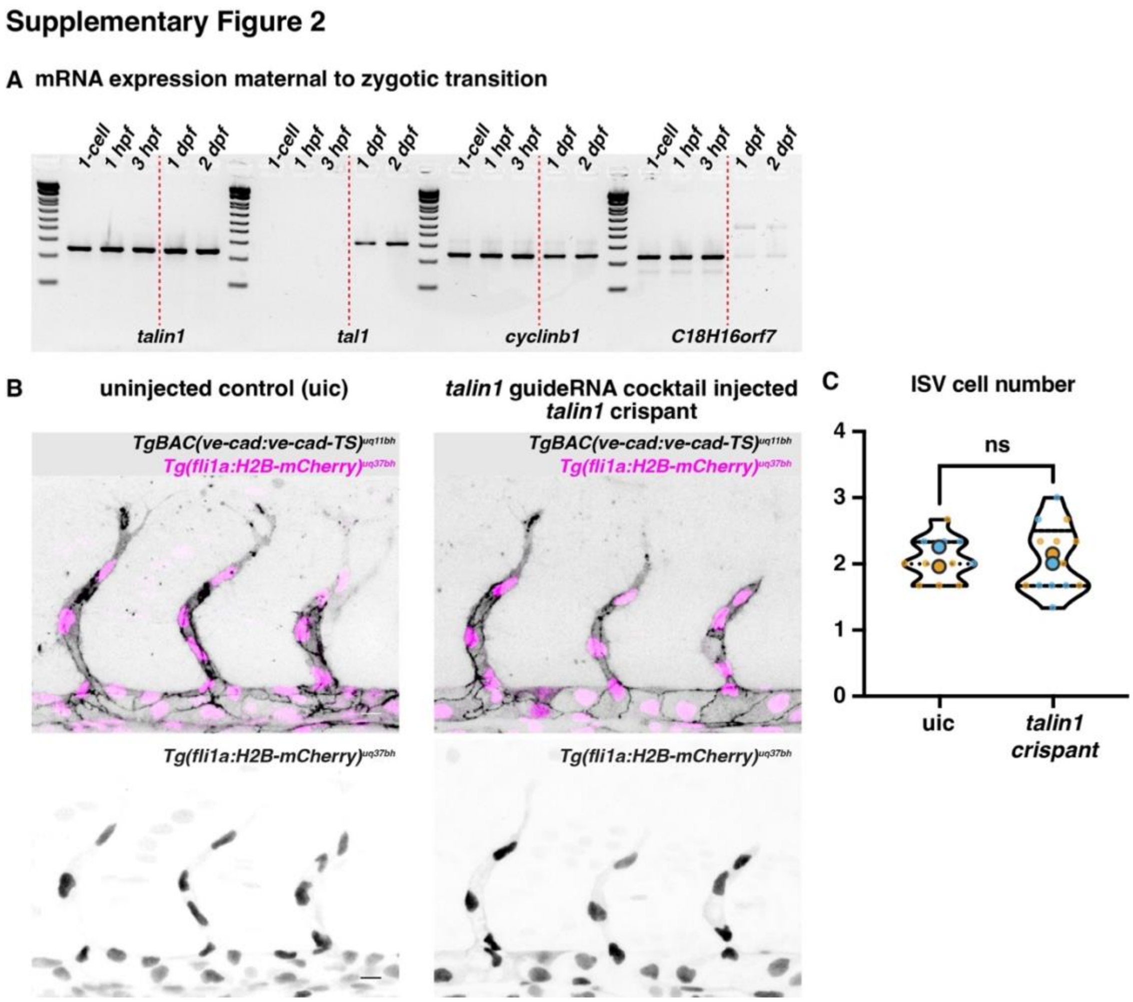

Fig. S2. Maternal contribution of Talin1 and EC number in sprouting ISVs

(A) RT-PCR analysis to determine *talin1* mRNA expression in lysates from 20 pooled wild-type embryos at 1-cell stage, 1 hpf, 3 hpf, 1 dpf and 2 dpf. Control genes include *cyclinb1* (maternally provided), C18H16orf7 (maternally provided, but not transcribed after zygotic transition) and *tal1* (not maternally provided). Red dashed line indicates maternal-zygotic transition. (B) Max projection of uninjected control and *talin1 crispant* embryo at 30 hpf showing EC nuclei in magenta (top) and grey (bottom). (C) Quantification of EC number in the ISVs at 30 hpf, 2 replicates, n=11 uninjected embryos and n=14 *talin1 crispants*. Replicate averages are depicted by large circles. Unpaired t-tests revealed no significant (ns) difference. Smaller circles present individual data points of each replicate (colour matched).

Development • Supplementary information

Supplementary Figure 3

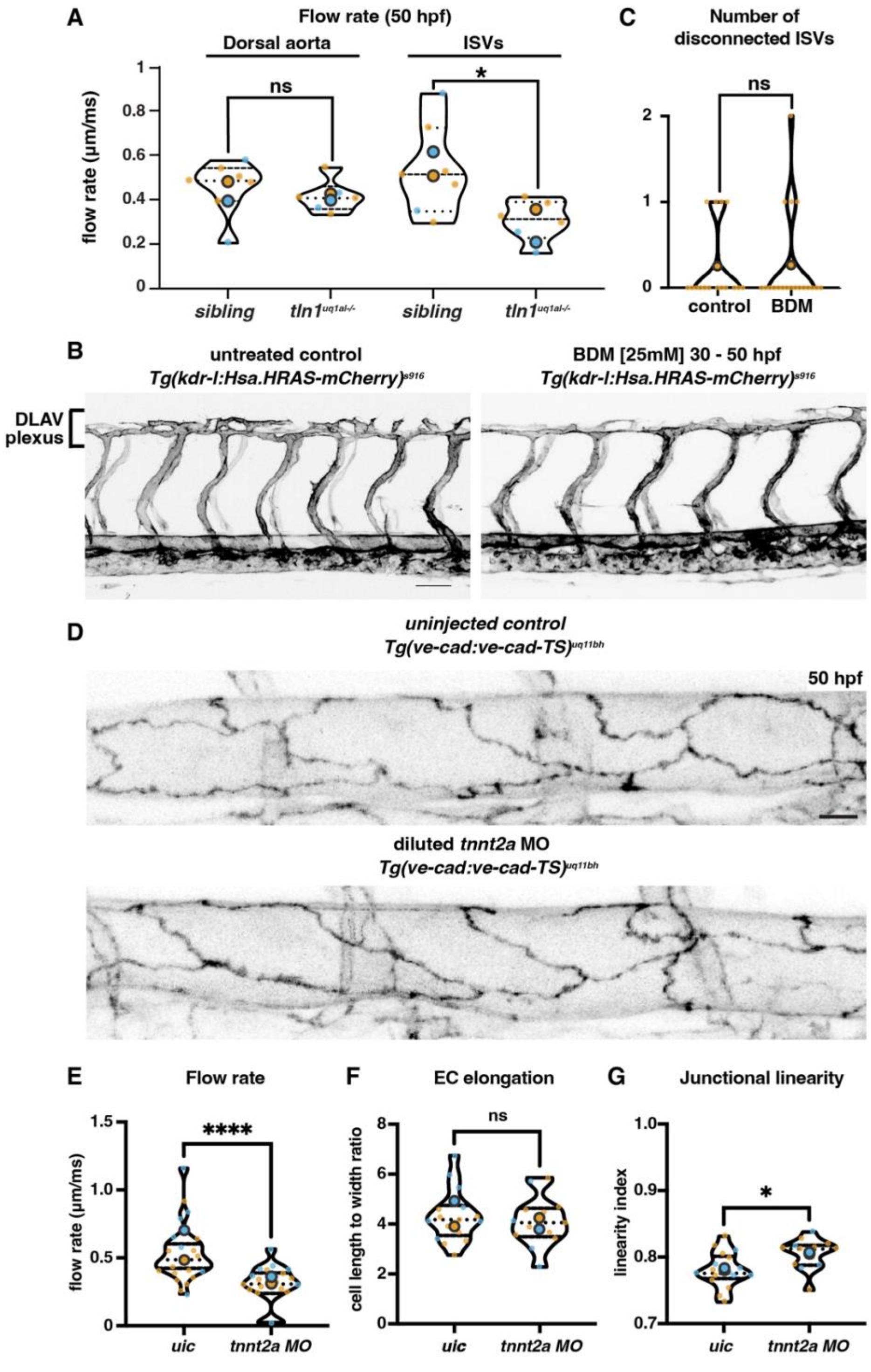

Fig. S3. Impact of reduced blood flow on vessel morphogenesis and EC elongation

(A) Flow rate (µm/ms) in the DA and ISVs of *tln1*$^{uq1al-/-}$ mutants and siblings at 50 hpf showing a significant reduction in flow only in the ISVs of mutants at this stage, 2

replicates, n=7 siblings and n=6 *tln1*$^{uq1al-/-}$ mutants. Unpaired t-test *p<0.05 (B) Max projection of the trunk vasculature in a BDM [25mM] treated and untreated control embryo at 50 hpf, revealing loss of DLAV development upon BDM treatment (blood flow loss). DLAV in control indicated by bracket. Scale bar = 50 µm. (C) Quantification of the number of disconnected ISVs across 6 body segments in BDM [25mM] treated and untreated control embryos, showing that flow loss does not induce ISV regression, 1 replicate n=16 control and n=19 BDM treated embryos, Mann-Whitney test revealed no significant (ns) difference. (D) Dorsal aorta of an uninjected control and diluted *tnnt2a* MO injected embryo at 50 hpf, expressing VE-cadherin-TS. Scale bar = 10µm (E) Quantification of flow rate (µm/ms) in the DA of uninjected control versus diluted *tnnt2a* MO injected embryos, n=2 replicates, n=21 controls and n=18 diluted *tnnt2a* MO, Mann-Whitney test ****p<0.0001 (F) Quantification of EC elongation in the DA, n=17 controls and n=13 diluted *tnnt2a* MO, unpaired t-test no significant (ns) difference (G) Quantification of junctional linearity of ECs in the DA, n=17 controls and n=13 diluted *tnnt2a* MO, unpaired t-test *p<0.05. In all graphs replicate averages are depicted by large circles. Smaller circles present individual data points of each replicate (colour matched).

Development • Supplementary information

Supplementary Figure 4

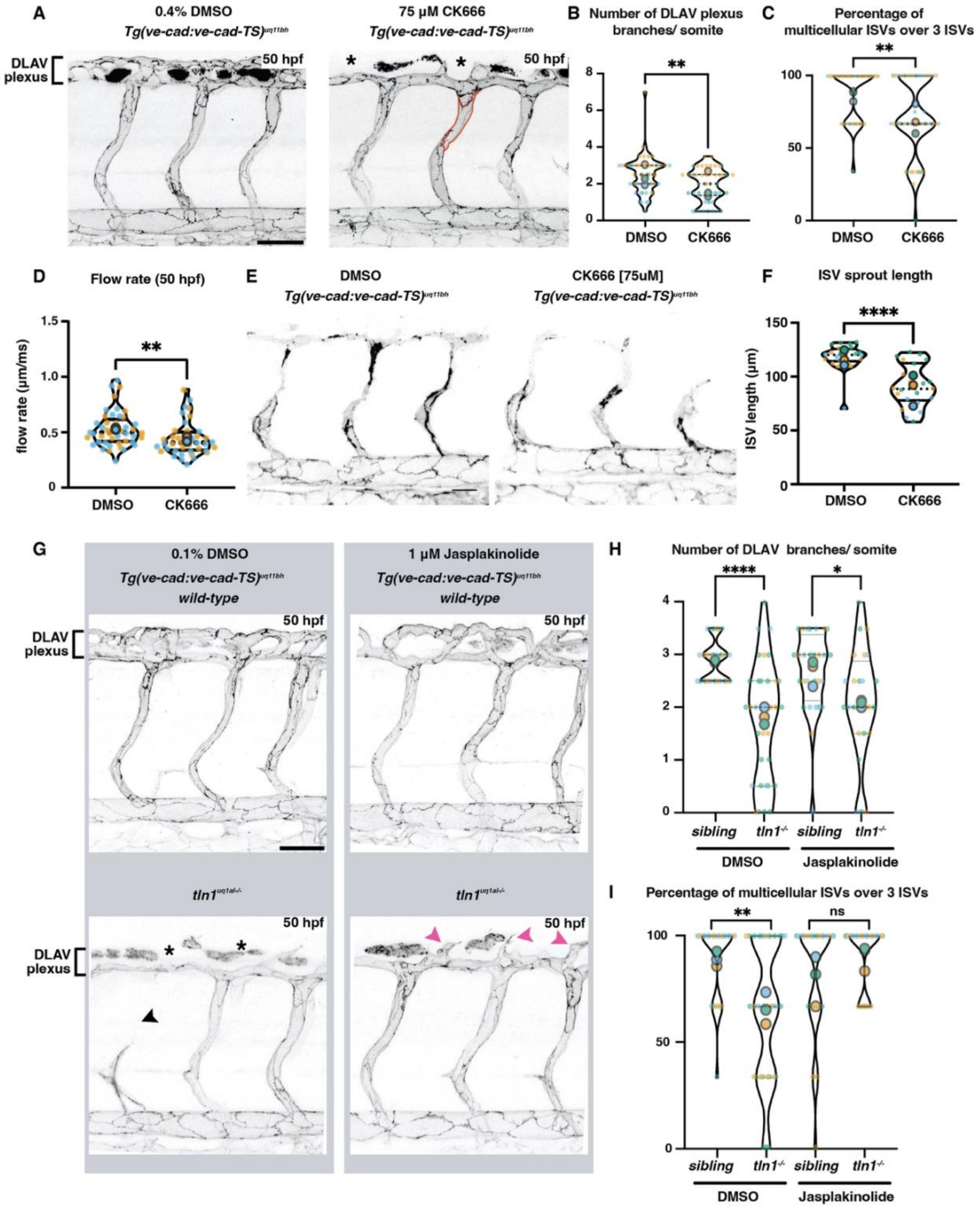

Fig. S4. Changes in vessel morphogenesis by CK666 and Jasplakinolide treatment

(A) CK666 treated wild-type embryos develop phenotypes that are similar to *tln1*$^{uq1al-/-}$ mutants, including compromised DLAV plexus formation (asterisk) and the more frequent occurrence of immature unicellular tubes (arrowhead). Scale bar = 50 µm (B) Quantification of the number of DLAV branches per somite, n=3 biological replicates, n=48 DMSO and n=48 CK666 treated embryos, Mann-Whitney test **p<0.01 (C) Quantification of the percentage of multicellular ISVs per 3 somites, n=3 biological replicates, n=48 DMSO and n=48 CK666 treated embryos, Mann-Whitney test **p<0.01 (D) Flow rate (µm/ms) in the DA of DMSO versus CK666 treated embryos at 50 hpf, 2 replicates, n=44 DMSO treated and n=40 CK666 treated embryos, unpaired t-test **p<0.01 (E) Live imaging of trunk vasculature of *Tg(ve-cad:ve-cad-TS)* positive wild-type embryos at 30 hpf. Embryos were treated with DMSO or CK666 [75µM] from 22-28 hpf. Scale bar = 50µm. (F) Quantification of ISV sprout length, showing a reduction in CK666 treated embryos, 3 replicates, n=29 DMSO treated and n=34 CK666 treated embryos, unpaired t-test ****p<0.0001 (G) Maximum projection of the trunk vasculature at 50 hpf, directly after treatment with either DMSO (control, left) or Jasp (right), showing more DLAV plexus branches in Jasp *tln1*$^{uq1al-/-}$ mutants (asterisk versus magenta arrowheads). Scale bar = 50µm. (H) Quantification of the number of DLAV branches per somite in DMSO versus Jasp treated embryos, n=3 biological replicates, n=25 siblings and n=33 *tln1*$^{uq1al-/-}$ mutants in the DMSO treated group and n=28 siblings and n=24 *tln1*$^{uq1al-/-}$ mutants in the Jasp treated group. For DMSO siblings versus *tln1*$^{uq1al-/-}$ mutants Mann-Whitney test ****p<0.0001. For Jasp siblings versus *tln1*$^{uq1al-/-}$ mutants Mann-Whitney test *p<0.05 (I) Quantification of the percentage of multicellular ISVs per 3 somites in DMSO versus Jasp treated embryos, n=3 biological replicates, n=25 siblings and n=33 *tln1*$^{uq1al-/-}$ mutants in the DMSO treated group and n=28 siblings and n=24 *tln1*$^{uq1al-/-}$ mutants in the Jasp treated group. In all graphs replicate averages are depicted by large circles. For DMSO siblings versus *tln1*$^{uq1al-/-}$ mutants Mann-Whitney test **p<0.01. For Jasp siblings versus *tln1*$^{uq1al-/-}$ mutants Mann-Whitney test with no significant (ns) difference. Smaller circles present individual data points of each replicate (colour matched).

Development • Supplementary information

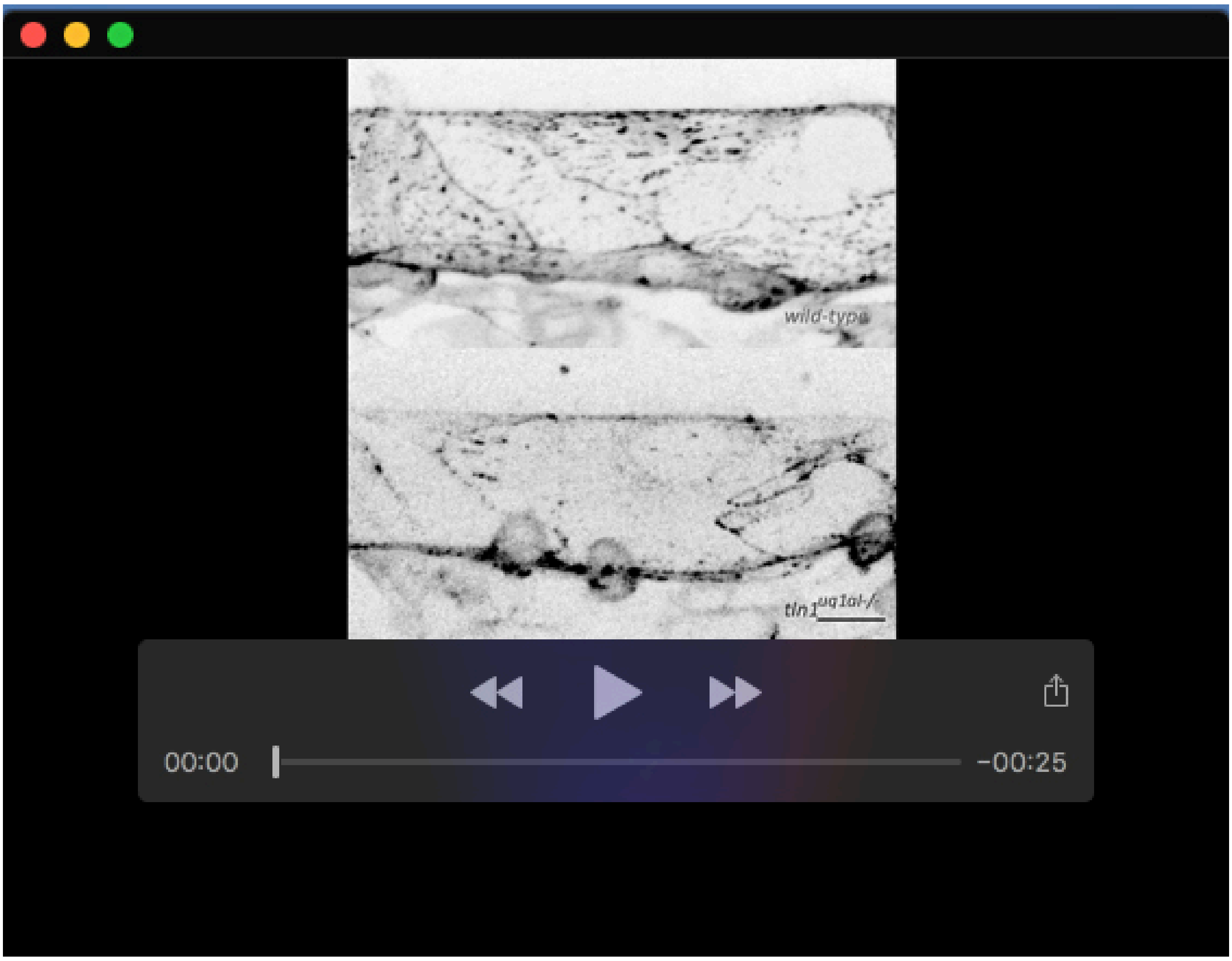

Movie 1. Time lapse movie showing endothelial Vinculin dynamics *in vivo*

Top: Dorsal aorta (DA) of a wild-type embryo, expressing *Tg(fli1ep:Vinculinb-eGFP)*^{uq2al}, showing dynamic Vinculin expression Focal Adhesions and cell-cell junctions over the course of the movie, from 50 hpf to 51 hpf.

Bottom: DA of a *tln1*$^{uq1al-/-}$ mutants, expressing *Tg(fli1ep:Vinculinb-eGFP)*^{uq2al}, showing a dramatic reduction of Focal Adhesions and diffuse cell-cell junctions expression, from 50 hpf to 51 hpf. Scale bar = 10µm.

Development • Supplementary information

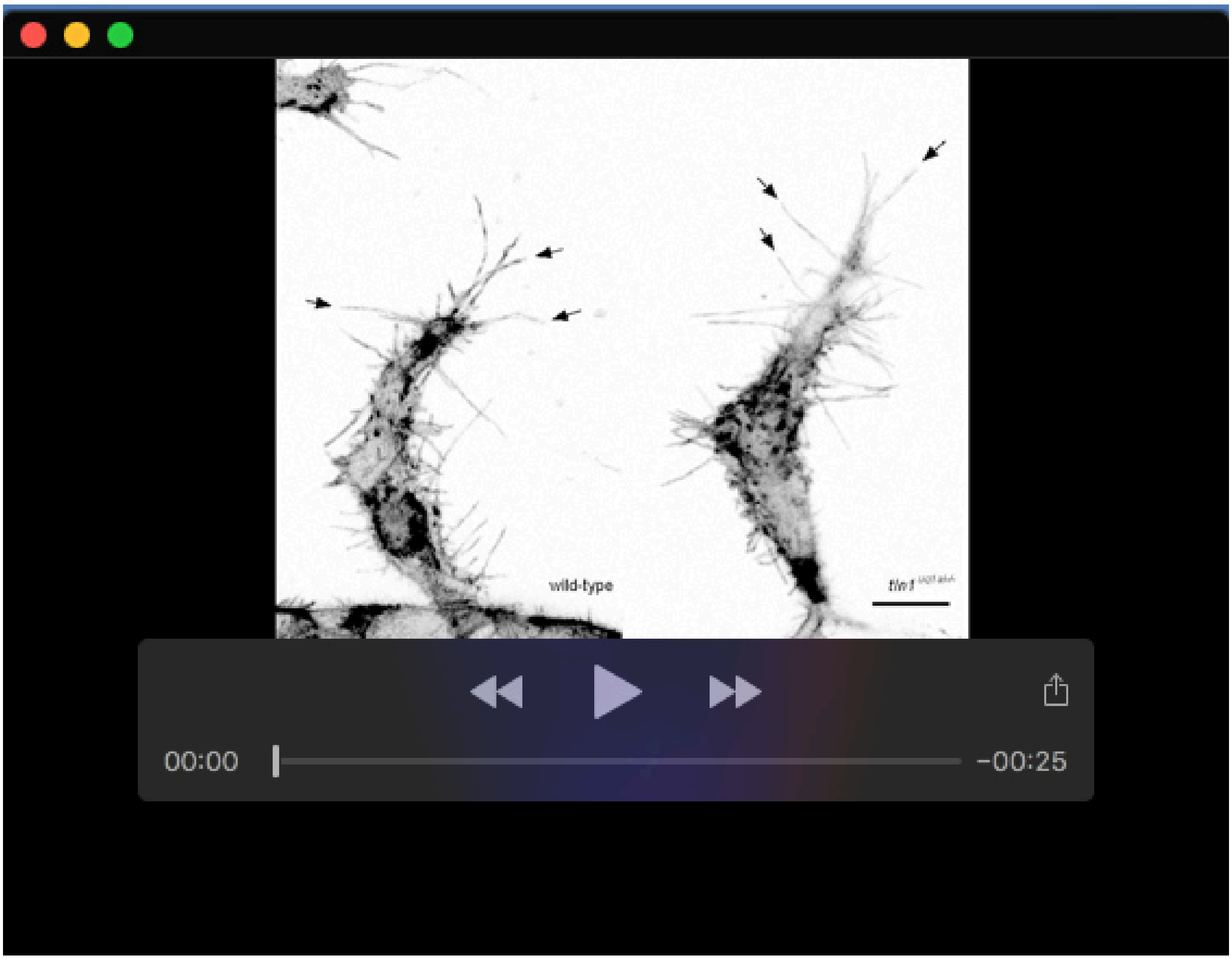

Movie 2. High-speed timelapse imaging of filopodia dynamics Time-lapse imaging of spouting ISVs in a wild-type embryo (left) and a *tln1*$^{uq1al-/-}$ mutant (right). To faithfully label filopodial extensions we utilised the membranous mCherry in ECs; *Tg(kdrl:Hsa.HRAS-mCherry)*^{s916}. Arrows indicate the emergence and retraction of filopodia. Movies were taken for 15 minutes starting at 26 hpf. Scale bar = 10μm.

Development • Supplementary information

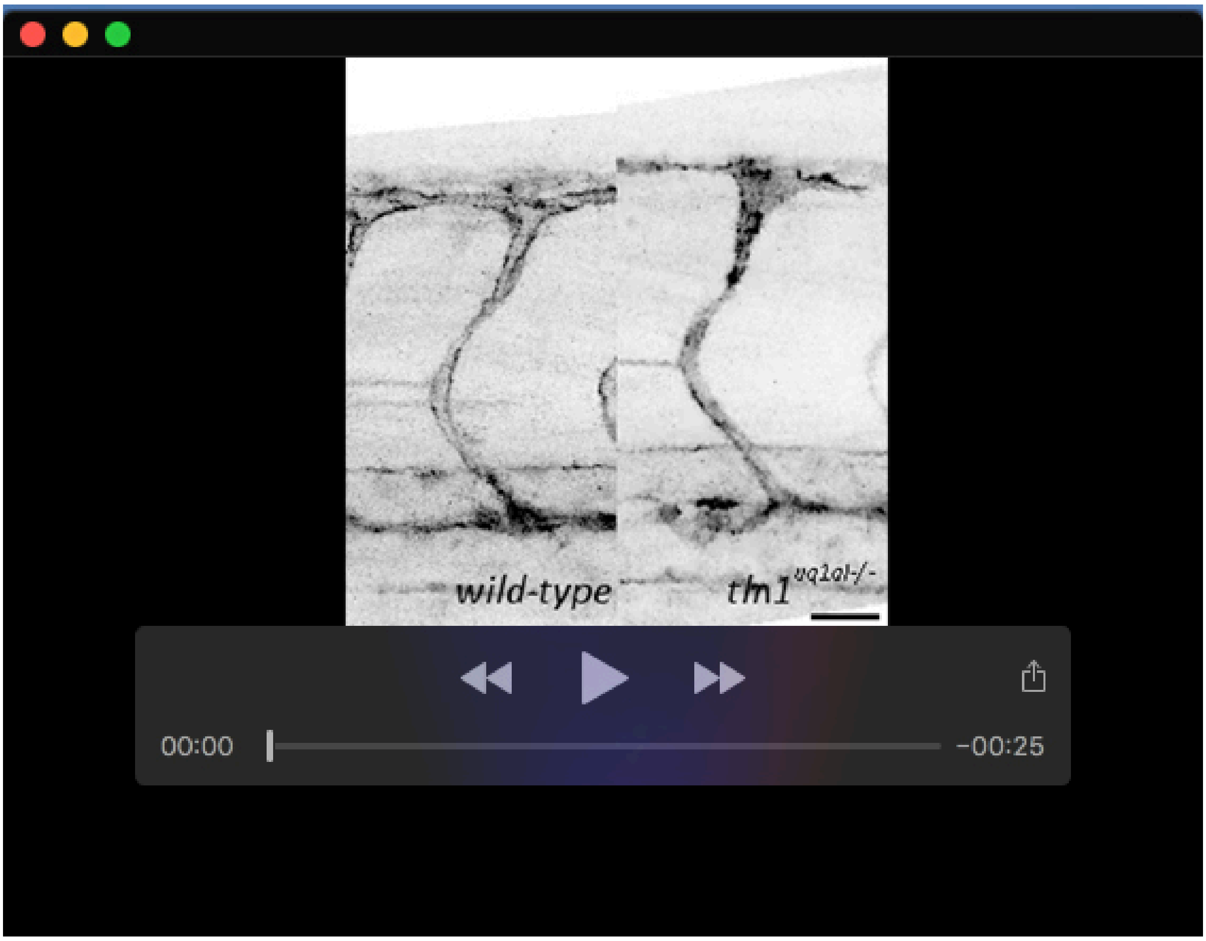

Movie 3. Time-lapse imaging during ISVs remodelling

Time-lapse imaging of ISVs in a wild-type embryo (left) and a *tln1*$^{uq1al-/-}$ mutant (right), showing disconnection of the ISV in the mutant. The endothelial F-actin marker line, *Tg(fli1ep:lifeact-eGFP)*^{uq3al}, was used to visualise the vasculature. Arrow indicates ECs disconnecting in the ISV of *tln1*$^{uq1al-/-}$ mutant. Movie taken from 30 hpf to 48 hpf. Scale bar = 25µm.

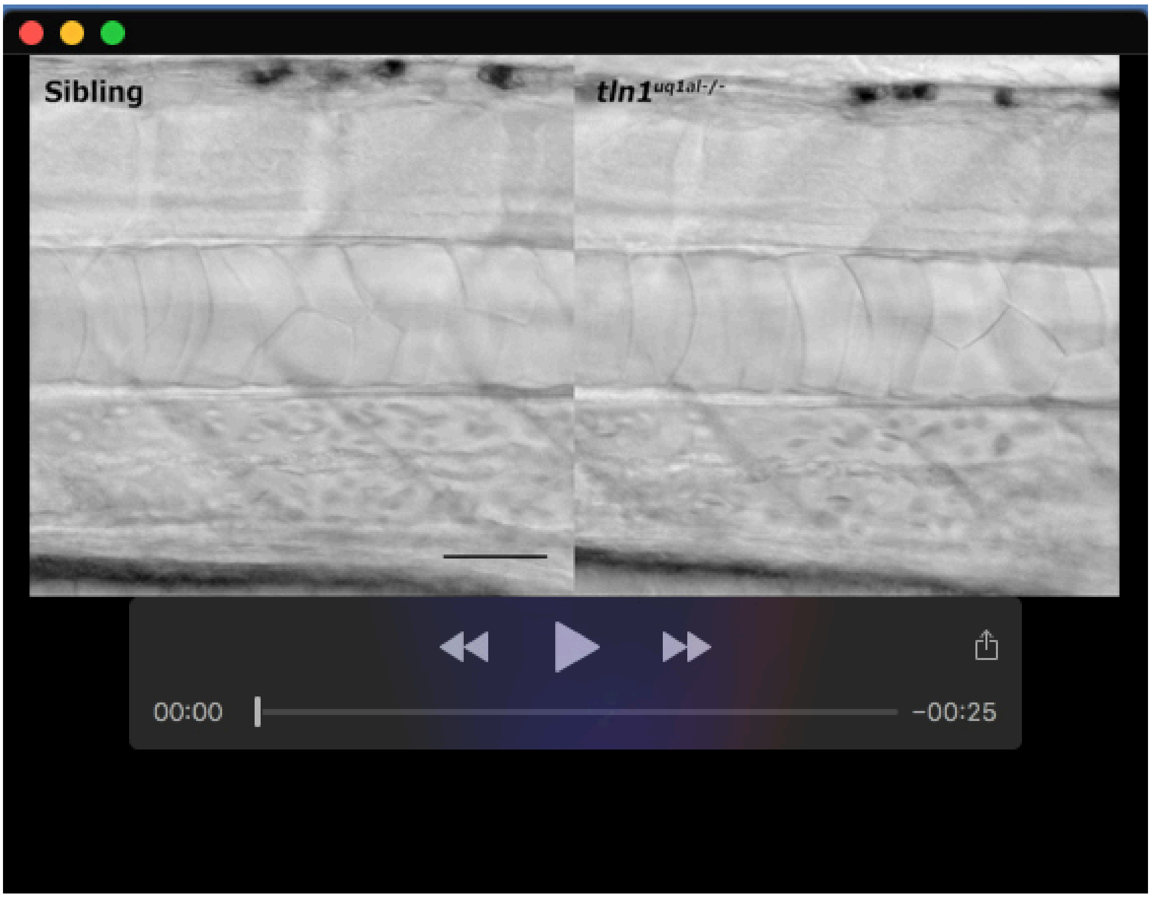

Movie 4. Recording of blood flow in the dorsal aorta

High speed recording of blood flow in dorsal aorta of a sibling (left) and a *tln1*$^{uq1al-/-}$ mutant embryo (right) at 50 hpf. Recording is shown at 250 frames/sec. Scale bar = 50µm.

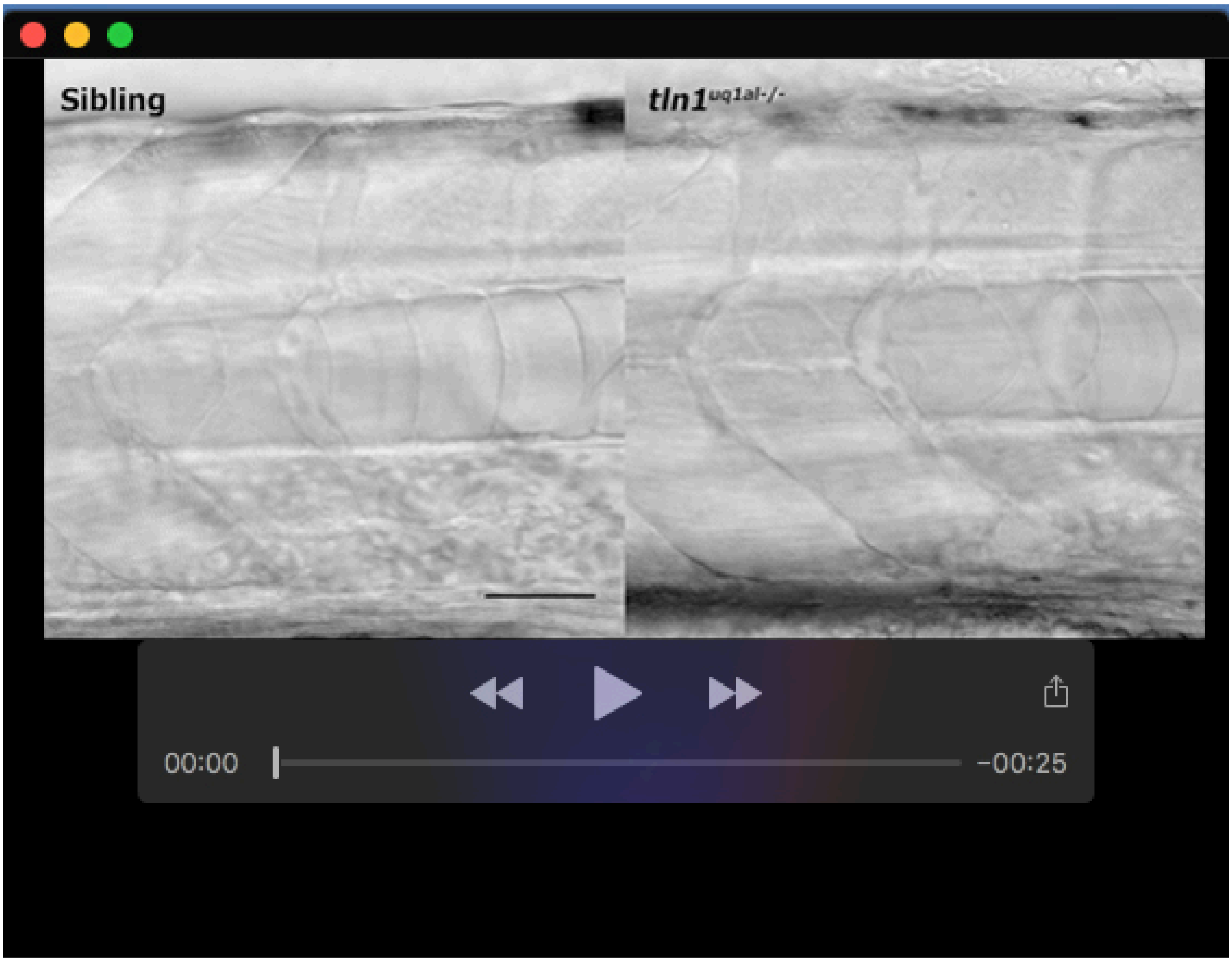

Movie 5. Recording of blood flow in the ISVs

High speed recording of blood flow in the ISVs of a sibling (left) and a *tln1*$^{uq1al-/-}$ mutant embryo (right) at 50 hpf. Recording is shown at 250 frames/sec. Scale bar = 50µm.

Development • Supplementary information

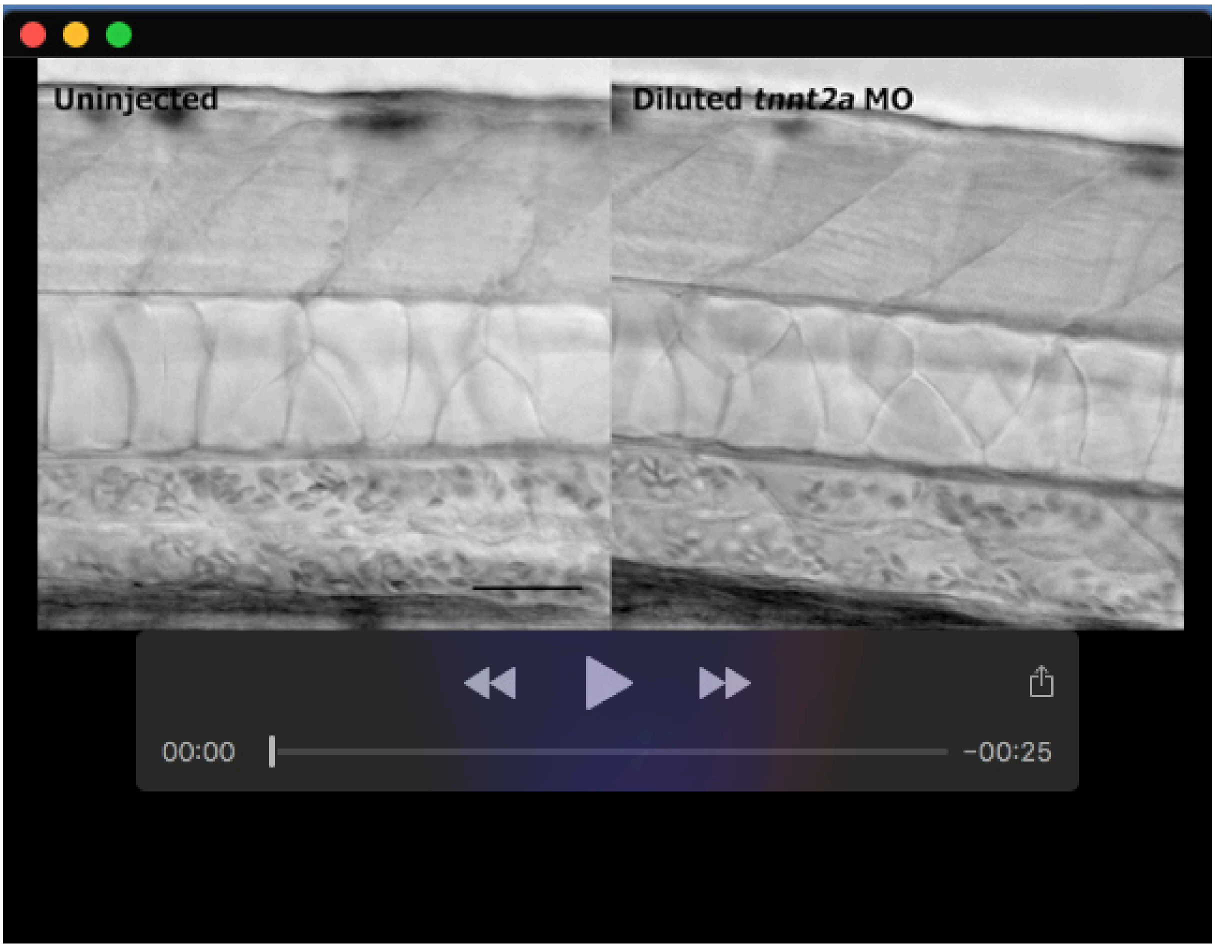

Movie 6. Reduced blood flow in diluted *tnnt2a* morpholino injected embryos

High speed recording of blood flow in the DA of a uninjected control (left) and a diluted *tnnt2a* MO injected embryo (right) at 50 hpf. Scale bar = 50µm.

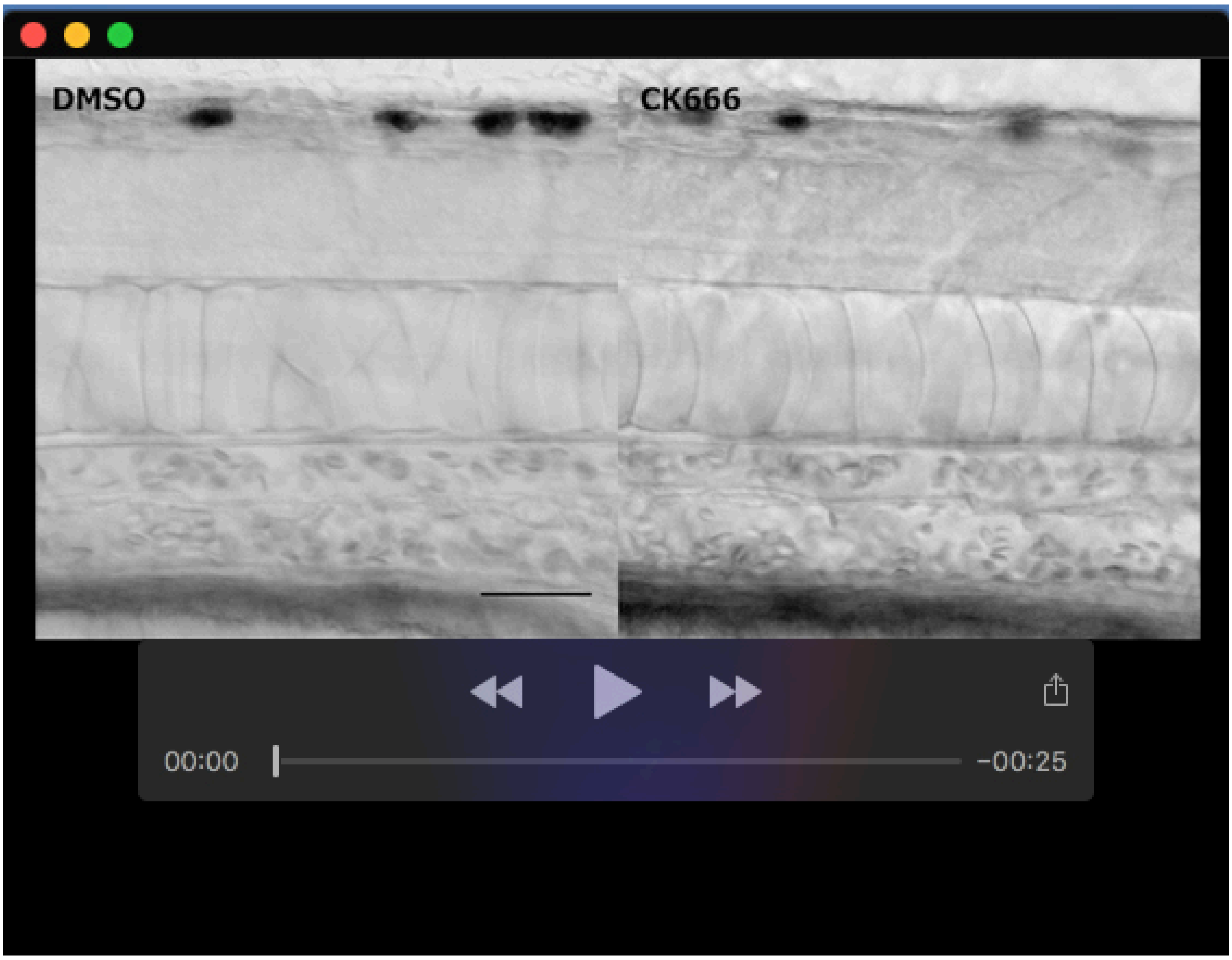

Movie 7. Reduced blood flow in diluted CK666 treated embryos High speed recording of blood flow in the DA of a DMSO control (left) and a CK666 [75 µM] treated embryo (right) at 50 hpf. Scale bar = 50µm.

Development • Supplementary information

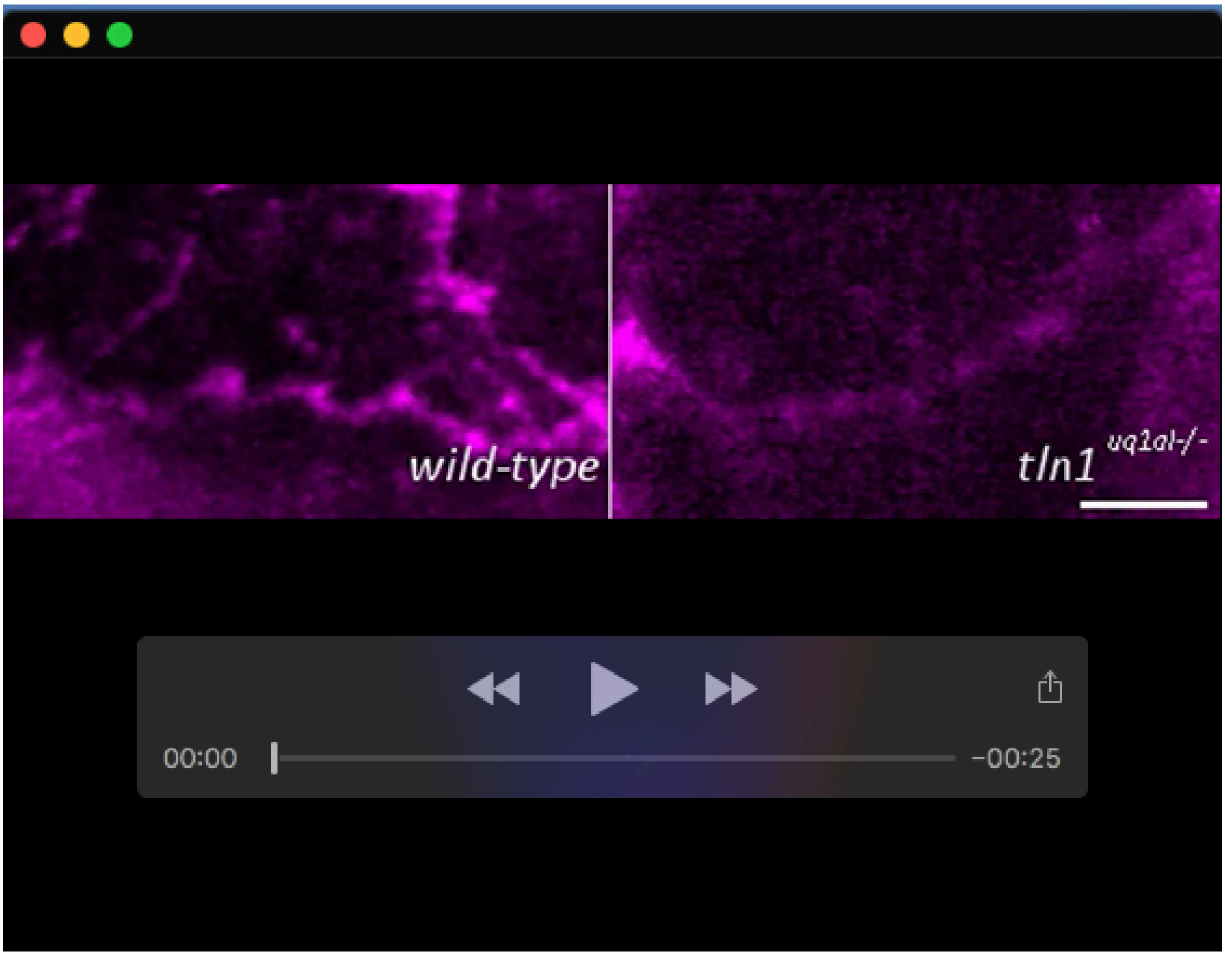

Movie 8. Impaired F-actin rearrangements in *tln1*$^{uq1al-/-}$ mutants Time-lapse imaging of cortical F-actin, visualised by *Tg(fli1ep:lifeact-eGFP)*^{uq3al}, near a cell-cell junction in a wild-type sibling (left) and a *tln1*$^{uq1al-/-}$ mutant (right). Movie was taken from 48 hpf to 49 hpf. Scale bar = 5μm.

Development • Supplementary information

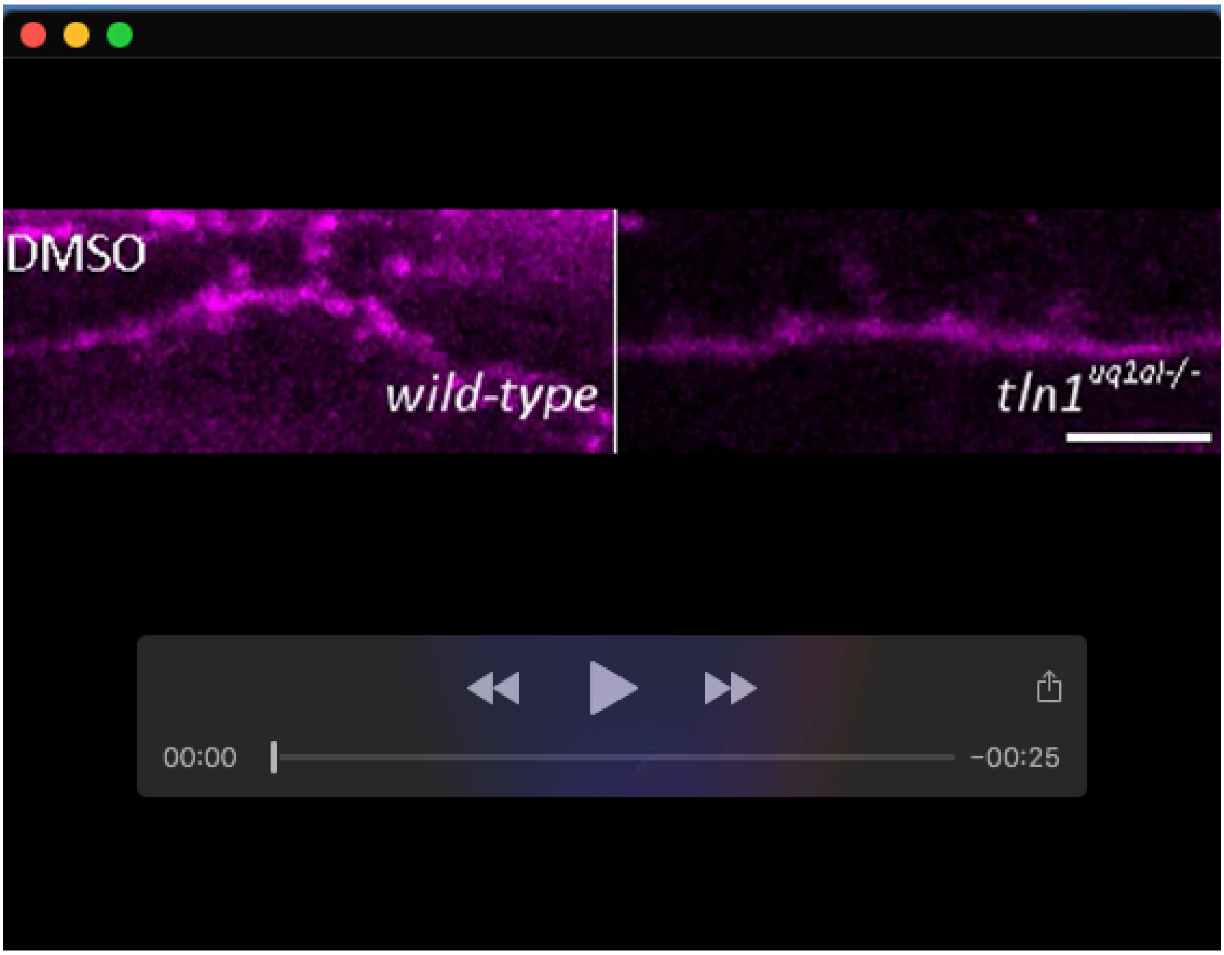

Movie 9. F-actin dynamics in DMSO treated sibling and *tln1^uq1al-/-^* mutant

Time-lapse imaging of cortical F-actin, visualised by *Tg(fli1ep:lifeact-eGFP)^{uq3al}*, near a cell-cell junction of a 0.1% DMSO treated wild-type sibling (left) and *tln1*$^{uq1al-/-}$ mutant (right). Embryos were treated for 5 hours prior to imaging. Movie was taken from 48 hpf to 49 hpf. Scale bar = 5µm.

Development • Supplementary information

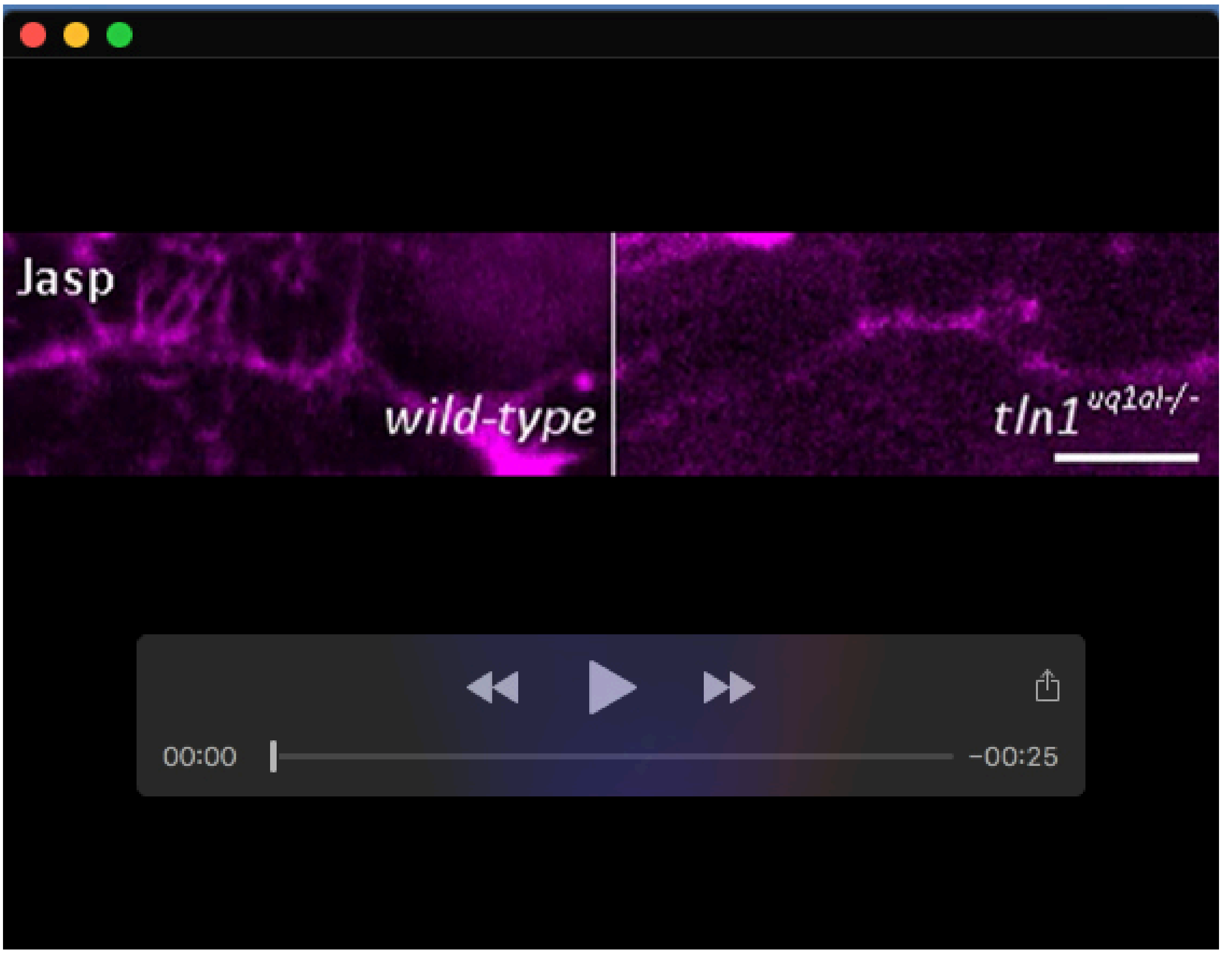

Movie 10. Rescue of F-actin rearrangements in Jasplakinolide *tln1$^{uq1al-/-}$* mutants

Time-lapse imaging of cortical F-actin, visualised by *Tg(fli1ep:lifeact-eGFP)^{uq3al}*, near a cell-cell junction of a Jasp [1µM] treated wild-type sibling (left) and *tln1$^{uq1al-/-}$* mutant (right). Embryos were treated for 5 hours prior to imaging. Movie was taken from 48 hpf to 49 hpf. Scale bar = 5µm.

Development • Supplementary information
