# Supplementary material for: Dynamically regulated focal adhesions coordinate endothelial cell remodelling in developing vasculature
Source: Development. 2022 Dec 12;149(23):dev200454. doi: 10.1242/dev.200454 (PMC10214536; doi:10.1242/dev.200454)
Supplement: Supplementary file 1 [file develop-149-200454_review_history.pdf]

Dynamically regulated focal adhesions coordinate endothelial cell remodelling in developing vasculature

Tevin C. Y. Chau, Mikaela S. Keyser, Jason A. Da Silva, Elysse K. Morris, Teodor E. Yordanov, Kinga P. Duszyc, Scott Paterson, Alpha S. Yap, Benjamin M. Hogan and Anne Karine Lagendijk
DOI: 10.1242/dev.200454

Editor: Steve Wilson

Review timeline

Original submission:	8 December 2021
Editorial decision:	18 January 2022
First revision received:	21 September 2022
Editorial decision:	12 October 2022
Second revision received:	14 October 2022
Accepted:	20 October 2022

Original submission

First decision letter

MS ID#: DEVELOP/2021/200454

MS TITLE: Dynamically regulated Focal adhesions coordinate endothelial cell remodelling in developing vasculature

AUTHORS: Chui-Ying (Tevin) Chau, Teodor Yordanov, Jason da Silva, Scott Paterson, Alpha Yap, Ben Hogan, and Anne Karine Lagendijk

I have now received all the referees' reports on the above manuscript, and have reached a decision. The referees' comments are appended below, or you can access them online: please go toBenchPress and click on the 'Manuscripts with Decisions' queue in the Author Area.

As you will see, the referees express considerable interest in your work, but have some criticisms and suggestions for improving your manuscript. If you are able to revise the manuscript along the lines suggested, I will be happy receive a revised version of the manuscript. Please also note that Development will normally permit only one round of major revision.

Please attend to all of the reviewers' comments and ensure that you clearly highlight all changes made in the revised manuscript. Please avoid using 'Tracked changes' in Word files as these are lost in PDF conversion. I should be grateful if you would also provide a point-by-point response detailing how you have dealt with the points raised by the reviewers in the 'Response to Reviewers' box. If you do not agree with any of their criticisms or suggestions please explain clearly why this is so.

© 2022. Published by The Company of Biologists under the terms of the Creative Commons Attribution License (https://creativecommons.org/licenses/by/4.0/).

Reviewer 1

Advance summary and potential significance to field

This manuscript by Chau, et al. aims to address the role of focal adhesions in remodeling the vasculature during development. This has been a phenomenon especially hard to monitor and demonstrate in vivo, and new tools developed in this manuscript are predicted to be important for the field. The manuscript nicely demonstrates the role of Talin in formation of FA in an animal, and demonstrates the requirement of FA for EC/junction elongation via F-actin rearrangement during vessel remodeling. The paper contains a number of elegant experiments and imaging, however a few questions remain before the manuscript can be recommended for publication.

Comments for the author

Major Comments:

- The Talin1 mutation could be a dominant negative and not a null. Is the protein degraded since it is predicted to be misfolded or could it be binding other focal adhesion proteins and sequestering their function, thus confounding these phenotypes? Western blotting for Talin1 protein would help to answer this.
- We see that talin mutants have blood flow and it is mentioned that cardiac output is "stable" until 2 dpf…

But does this mean the output is the same in talin mutants? Since blood flow can affect FA formation, this needs to be more thoroughly addressed and presented in data form. Perhaps measuring heart rate or blood flow rate at analogous time points of when other measurements are made would help answer this. The authors suggest that blood flow might be decreasing between 2-3 dpf? If this is the case, how is this progressive loss of flow separated from failed EC elongation at the analyzed time points?
- Along these same lines, is cardiac output affected in CK666-treated embryos & and is angiogenesis delayed similar to the Talin mutants at 30 hpf?
- Sprout length is reduced in talin mutants, which is hypothesized to be from EC elongation. Is there also a lack of EC proliferation/increased apoptosis or is cell number stable?
- In the DA (Fig 3G)- the cell shape is clearly different, but is there also a difference in cell number in the talin KO?

Minor Comments:

- How do you define biological replicate? Is it a clutch and each embryo is meant to be a technical replicate within that clutch?
- Are the mutant ISVs perfused at 50 hpf before they start regressing? Or are they never perfused at all?

Lack of perfusion can contribute to regression so please comment/address within the text.

Reviewer 2

Advance summary and potential significance to field

This manuscript by Chau et al., "Dynamically regulated Focal adhesions coordinate endothelial cell remodelling in developing vasculature" is the first to comprehensively examine the effects of focal adhesion defects on vascular morphogenesis and integrity in vivo, in a living organism with intact blood flow. The authors find that the endothelial cells (ECs) of zebrafish talin1 mutants exhibit mature focal adhesion loss, impaired F-actin organization, and reduced inter-EC junction linearization – leading to reduced EC elongation, and angiogenesis and vascular integrity defects. The authors determine that polymerized F-actin is required for EC elongation, and that pharmaceutical stabilization of F-actin partially rescues the EC remodeling and angiogenesis defects of talin1 mutants.

Vascular development and integrity defects (including EC actin network deficiency) in zebrafish talin1 mutants have been described previously (Wu et al., 2015). However, this manuscript

© 2022. Published by The Company of Biologists under the terms of the Creative Commons Attribution License (https://creativecommons.org/licenses/by/4.0/).

describes the vascular morphogenesis, focal adhesion, and EC junction defects of talin1 zebrafish mutants in much greater detail, and provides novel mechanistic insights into how focal adhesions and actin cytoskeletal dynamics promote EC elongation and blood vessel maturation in vivo. The authors of this study also develop and introduce a novel transgenic zebrafish model, Tg(fli1ep:Vinculinb-eGFP)^{uq2al}, that can be used to examine EC focal adhesion dynamics in vivo. For these reasons, this manuscript it is of potential significant interest to the vascular development and vascular biology communities.

Comments for the author

Overall, the study is interesting, the experiments are generally well performed, the manuscript is well written, and the images and movies are of good quality. However, additional experimentation and clarification is required to address some issues, as described below.

Major Issues:

The authors develop a novel talin1 mutant zebrafish line, tln1^{uq1a1}, with a mutation in the F1 FERM subdomain, which is predicted by in silico analysis to disrupt integrin binding. This prediction should be experimentally validated and/or the authors should provide additional evidence that tln1^{uq1a1} is a null allele. Are overall Tln1 protein levels changed tln1^{uq1a1}-mutants?

Previous studies have suggested that interplay between EC elongation, proliferation, and polarized migration is required for proper angiogenesis, multicellular tube formation, and blood vessel maturation. The authors should determine if EC proliferation or overall migration (e.g., nuclear movement) are altered in the ISVs and dorsal aorta of tln1-mutants.

The authors perform transplant experiments to determine if Talin1 functions in an EC-autonomous manner to regulate EC elongation. However, it is unclear how many non-endothelial donor cells are also being transplanted into recipients, and if those cells might also contribute to the observed vascular phenotypes. (How mosaic are recipient embryos following transplant? I.e. do the cells surrounding labeled ECs also originate from tln1-mutant donors?) To further substantiate their claim that Talin1 functions EC autonomously, the authors should determine either (i) if the ECs of wild type donor embryos elongate properly when transplanted into tln1-mutant recipients, or (ii), if expressing wild type talin1 specifically in the ECs of tln1-mutants rescues their EC-elongation and/or ISV angiogenesis defects.

Minor Comments:

Given that tln1-mutants exhibit haemorrhaging in the brain, the authors should determine if brain vascular development is also perturbed in tln1-mutants.

In embryos transplanted with tln1-mutant cells, do only the mutant ECs demonstrate reduced elongation, or do neighboring wild type recipient ECs also share this phenotype? This should be examined.

Given that EC elongation and junction linearity in tln1-mutants is partially rescued following Jasplakinolide treatment (actin stabilization), the authors should indicate if the hemorrhage phenotype is also rescued.

Reviewer 3

Advance summary and potential significance to field

Chau and colleagues analyze the requirement of Talin-1 during blood vessel formation and remodeling during early zebrafish development. To this end, they generate a targeted mutation of talin-1. They also generate a transgenic reporter line to visualize Vinculin in endothelial cells. Using these tools the authors show that talin-1 mutant lack focal adhesions. They further show that Talin-1 is required for efficient sprouting junctional rearrangement during ISV formation, blood vessel maintenance and endothelial cell elongation.

© 2022. Published by The Company of Biologists under the terms of the Creative Commons Attribution License (https://creativecommons.org/licenses/by/4.0/).

Analysis of genetic mosaics demonstrates that Talin-1 is required cell-autonomously. Finally, to identify the cellular mechanisms which are affected in talin-1 mutants, they image F-actin and manipulate F-actin dynamics by pharmacological intervention. Whereas, inhibition of Arp2/3 mimics aspects of the talin-1 mutant phenotype, stabilization of F-actin fibers by treatment with jasplakinolide can rescue the mutant phenotype.

Overall, the authors use a new model to study focal adhesions during vascular morphogenesis and present a set of very interesting findings linking focal adhesions to junctional dynamics underlying vascular tube formation.

Comments for the author

A few issues need to be addressed.

1) Characterization of the talin-1 mutant allele. The authors suggest that the generated talin-1 mutant is a null allele, but do not present data that would support this view. A potential dominant-negative effect could be determined by comparing wild-type, heterozygous and homozygous talin-1 mutants. Furthermore injection of the mutant RNA should not cause any defects observed in the homozygous mutant.

2) RNA levels of the other talin paralogs does not significantly change in talin-1 mutants. However, the presence of these paralogs raises the issue of functional redundancy. Do the authors have any information on the expression patterns of the paralogs? Later on the authors mention that maternal talin-1 contribution may partially rescue loss of zygotic Talin-1. Do the authors have concrete information on maternal talin-1 RNA or protein. Are talin-1 mutants homozygous viable/fertile? In which case, the authors could test maternal contribution in mz talin-1 embryos.

3) Figure 1: The loss of Vinculin-GFP clusters in talin-1 mutants is quite dramatic. It would be important to have a second independent to demonstrate that these clusters are indeed focal adhesions. The authors should try antibodies against FAK and P-Paxillin, which have previously been shown to work in heart of zebrafish embryos.

4) Figure 1: panel D-F show Vinculin clusters at cell junctions, which are gone in talin-1 mutants. Do the authors see similar changes with junctional markers such as the VE-Cad-TS transgene? Panel G: Along the same lines. Is the cell-cell junction narrowed in a comparable fashion in the VE-cad-TS line?

5) Figure 2: focal adhesions are thought to play an important role in cell migration. In agreement with this notion, talin-1 mutant show reduced sprouting speed. Focal adhesions may also serve as a clutch to support filopodial elongation. The authors provide still pictures, which show that filopodial number and length are only mildly affected – if at all. As filopodia are very dynamic cellular processes, it would be important to perform time-lapse imaging to determine, whether filopodial dynamics are disturbed in talin-1 mutants.

6) Figure 3: panels A-F. Could the defects in DLAV remodeling be caused by lack of blood flow in the ISV/DLAV? Did the authors check for blood flow in these vessels. Is the disconnection within the ISV preceded by loss of blood flow – if not – does it lead to hemorrhages?

7) Figure 3panels G-I and Figure 5: mutant ECs in the DA appear wider and their junctions less straight compared to siblings. The authors argue that cell elongation requires mechanical forces generated by the F-actin cytoskeleton, which is linked to focal adhesions. While it is well established that the actin cytoskeleton is a major contributor to endothelial cell shape, I do not understand how inhibition of Arp2/3 addresses this point. From the data shown, it appears that many of the defects observed in talin-1 mutants may ultimately be caused by junctional destabilization. Since this group has established the VE-cadherin-based tension sensor in zebrafish, I wonder whether they have examined junctional tension in talin-1 mutant. This would be much more direct evidence that loss of Talin1 affects mechanical forces in the DA.

8) Figure 6: The interpretation of the jasplakinolide may not be as straight forward as the authors suggest.

© 2022. Published by The Company of Biologists under the terms of the Creative Commons Attribution License (https://creativecommons.org/licenses/by/4.0/).

Besides stabilizing F-actin fibers, it has been shown to enhance F-actin nucleation and polymerization in cell culture (e.g. Bubb et al., Journal of Biological Chemistry (2000)). Therefore, it is possible (or even more likely) that the phenotypic rescue of talin-1 mutants by jasplakinolide is mediated by enhanced F-actin polymerization.

Minor points:

"To our knowledge, this represents the first in vivo model that reports FA dynamics in ECs of live vasculature". (p8) This statement is more appropriate for the Discussion section.

"… anchorage of filopodia to the ECM occurs via FAs ". (p8) This statement should be referenced

" … EC elongation is involved in the transformation of pre-mature unicellular ISVs …" . (p9) unicellular ISVs are not pre-mature.

References 43 (Bentley) 44 (Perryn) do not support the statement about multicellular tube formation.

What do the authors mean by "Guided" EC elongation? (p9)

"FAs are essential to facilitate EC elongation" (p10/11)– I am not sure what the authors mean by "facilitate".

First revision

Author response to reviewers' comments

Detailed Response to Reviewer's comments

Reviewer #1: This manuscript by Chau, et al. aims to address the role of focal adhesions in remodeling the vasculature during development. This has been a phenomenon especially hard to monitor and demonstrate in vivo, and new tools developed in this manuscript are predicted to be important for the field. The manuscript nicely demonstrates the role of Talin in formation of FA in an animal, and demonstrates the requirement of FA for EC/junction elongation via F-actin rearrangement during vessel remodeling. The paper contains a number of elegant experiments and imaging, however a few questions remain before the manuscript can be recommended for publication.

Questions:
1. The Talin1 mutation could be a dominant negative and not a null.
Is the protein degraded since it is predicted to be misfolded or could it be binding other focal adhesion proteins and sequestering their function, thus confounding these phenotypes? Western blotting for Talin1 protein would help to answer this.

We thank the reviewer for these questions regarding the nature of the *talin1* mutation and how it impacts Talin1 protein expression and function.

We agree with the reviewer that Western blot analysis is a superior method to evidence degradation on our mutated Talin1 protein. This has also been pointed out by the two other reviewers
We now provide **new Western Blot data in Figure 1 of the manuscript (panel D),** revealing a marked reduction in Talin1 protein expression at 3 dpf. Based on this new data we conclude that the in-frame deletion in the FERM domain results in degradation of the mutated protein. We have performed three replicates of this experiment on three independent clutches of embryos. The results for the other two other replicates is included here as **Figure 1 for Reviewers**.

© 2022. Published by The Company of Biologists under the terms of the Creative Commons Attribution License (https://creativecommons.org/licenses/by/4.0/).

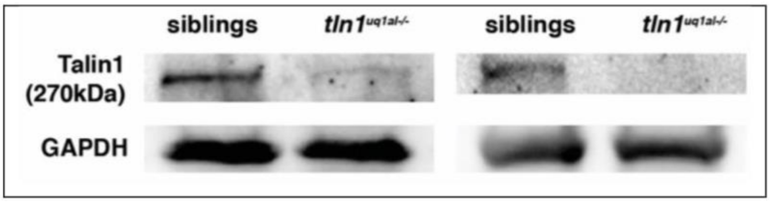

Figure 1: Replicate Western blots for Talin1 (270kDa) in lysates from sibling and *tln1*$^{uq1al-/-}$ mutant embryos at 3 dpf

Further, to show that the mutation in *tln1*^{uq1al} does not induce a dominant negative phenotype, we have taken brightfield images of *tln1*$^{uq1al-/-}$ mutants and siblings at both 50 hpf and 5 dpf. These images show that heterozygotes are indistinguishable from homozygous wild-type animals at these stages **(Figure 2 for Reviewers)**.

We further also would like to note that *tln1*$^{uq1al+/-}$ heterozygote adults are viable and that genotypes have been distributed at expected Mendelian ratios across all our adult generations of this line that have been bred over the past ~5 years.

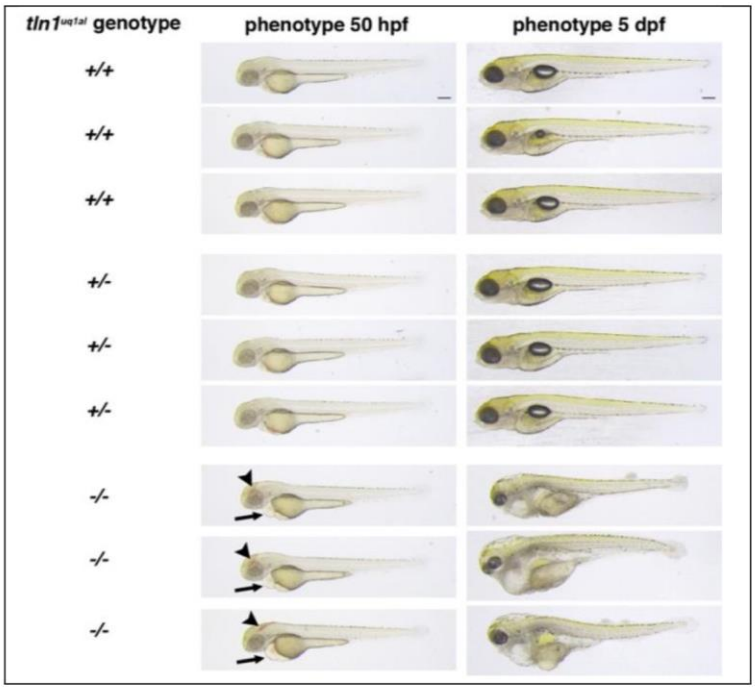

Figure 2: Representative brightfield images of each *tln*^{uq1al} genotype (3 embryos per genotype). Homozygous wild-type embryos (top) are indistinguishable from heterozygotes (middle) at both 2 dpf and 5 dpf, whilst homozygous mutant embryos present with cranial haemorrhages (arrowheads) and cardiac edema (arrows) at 2 dpf. At 5 dpf, the overall phenotype of homozygous mutants has worsened, and embryos are not viable past this developmental stage. Scale bar = 100µm

2. Is cardiac output is the same in talin mutants? The authors suggest that blood flow might be decreasing between 2-3 dpf? If this is the case, how is this progressive loss of flow separated from failed EC elongation at the analyzed time points?

We thank the reviewer for emphasizing the relevance of blood flow for overall EC biology. Indeed, vessels of 2 dpf *tln1*$^{uq1al-/-}$ mutants are still experiencing flow pressure. We have now performed additional experiments to precisely determine differences in flow velocity. Quantifications at 50 hpf revealed that there was a significant reduction of flow in ISVs at this stage, whilst flow in the DA was not significantly changed. We have added this **new data to new Supplementary Figure 3 (panel A)**.

© 2022. Published by The Company of Biologists under the terms of the Creative Commons Attribution License (https://creativecommons.org/licenses/by/4.0/).

To separate flow from Talin1 loss, we have performed the following experiments:

1. Cellular transplantation analysis presented in **Figure 4** of the original manuscript. Here we placed *tln1*$^{uq1al-/-}$ mutant cells in wild-type animals. Since these wild-type animals develop normally, cardiac output is not affected and our observations in terms of EC elongation are solely due to loss of Talin1 function and not due to changes in blood flow. Analysis at 3 dpf have only been based on transplanted ECs that are experiencing wild-type flow.

2. For this revised version of the manuscript we have performed additional transplant experiments, whereby we have visualised all transplanted cells (not just ECs), by injecting the donors with cascade blue dye at 1-cell stage. We aimed to examine small grafts that were EC restricted, i.e with no cascade blue expression in surrounding cell types. Such EC restrictive grafts are extremely rare and thus we first established an alternative method of Talin1 loss of function that would allow us to transplant Talin1 deficient cells at a higher rate. To do this we utilised a previously reported guideRNA cocktail knockout approach[1].

We designed four individual guideRNAs, all targeting *talin1* genomic sequence. We transcribed and pooled these guides for injection into wild-type embryos at 1 cell stage to disrupt Talin1 transcription/translation into functional protein. We further refer to *talin1* guide injected embryos as "*talin1 crispants*".
To validate the efficiency of this approach, we first scored the number of embryos that were phenotypically similar to *tln1*$^{uq1al-/-}$ mutants at 50 hpf. From two independent experiments we determined that ~80% of *talin1 crispants* phenocopied *tln1*$^{uq1al-/-}$ mutants; developing bleedings in the brain and mild cardiac oedema. The remaining 20% of embryos were either less severe, characterised by focal bleedings in the brain without prominent cardiac phenotypes, or more severe, developing severe cardiac oedema and complete loss of circulation.
We next determined DNA cutting efficiency of each of the four guides by amplifying the targeted amplicon. We performed this analysis on 8 injected embryos form each of the two independent injection rounds and identified that each guide induced an overall lack of a distinctive amplicon PCR bands and the appearance of additional amplicons of different sizes. These results indicate CRISPR/Cas9 induced changes to the genomic region targeted by these guideRNAs.
This extensive validation has been added to **Supplementary Figure 1 (panels D-F)**.

We transplanted cells from double transgenic *talin1 crispant* donors. These carried both *TgBAC(ve-cad:ve-cad-TS)*, labelling VE-cadherin at cell-cell junctions and *Tg(fli1ep:nls- mCherry),* marking the EC nuclei. Cells from these donors were transplanted into *TgBAC(ve-cad:ve-cad-TS)* wild-type embryos. This allowed us to monitor EC shape of both Talin1 deficient donor ECs and neighbouring wild-type ECs.
We identified EC restricted grafts in n=8 embryos from 2 independent rounds of transplantations. We quantified EC shape of Talin1 deficient donor cells versus neighbouring wild-type ECs at 2 and 3 dpf. This analysis confirmed our previous findings that Talin1 is required cell-autonomously for EC elongation and is **added to the manuscript in Figure 4 (panels E-G).**

3. Finally, although blood flow velocity was not significantly different in the DA of *tln1*$^{uq1al-/-}$ mutants at 50 hpf **(new Supplementary Figure 3A)**, we sought to directly test whether reducing blood flow in wild-type animals would inhibit EC elongation. To examine this, we injected a suboptimal dose of *tnnt2a* morpholino (referred to as diluted *tnnt2a* MO) in wild-type embryos. This approach was recently published by Vignes et al[2]. We validated here that injection of ~0.15 ng of this morpholino reduced blood flow velocity. Notably, EC elongation was not affected in the DA of diluted *tnnt2a* MO injected embryos, and we made the interesting observation that reduced flow induced linearisation of the EC junctions. **These new observations have been added to the manuscript in new Supplementary Figure 3 (panels D-G).**

3. Is cardiac output affected in CK666-treated embryos and is angiogenesis delayed similar to the Talin mutants at 30 hpf?

In response to the reviewer's request to expand our analysis of CK666 treated embryos, we have quantified blood flow velocity in the DA of embryos treated with either DMSO or CK666 [75µM] from 24 hpf till 50 hpf. Flow velocity measurements were performed at the end of the treatment (50

© 2022. Published by The Company of Biologists under the terms of the Creative Commons Attribution License (https://creativecommons.org/licenses/by/4.0/).

hpf). This analysis revealed a reduction in flow velocity in CK666 embryos **(new Supplementary Figure 4, panel D)**. Notably, this reduction is comparable to that measured in diluted *tnnt2a* MO injected embryos, where EC elongation was not affected **(New Supplementary Figure 3, panel E).** This data, together with transplant data **(Figure 4)**, supports the conclusion that EC elongation is a consequence of Talin1 loss which inhibits actin polymerisation and that this is independent of changes in blood flow.

We thank the reviewer for the question whether CK666 would also inhibit sprouting angiogenesis. We have now performed CK666 [75µM] and DMSO treatments of wild-type embryos from 22 hpf till 28 hpf. We subsequently imaged ISV sprouts and quantified sprout length at 29-30 hpf. This analysis revealed a significant reduction in sprout length when Arp2/3 assisted actin polymerisation is inhibited. This data is added to the manuscript in **new Supplementary Figure 4 (panels E and F)**.

4. Sprout length is reduced in talin mutants, which is hypothesized to be from EC elongation. Is there also a lack of EC proliferation/increased apoptosis or is cell number stable?

We thank the reviewer for this important question. To specifically interrogate EC number in angiogenic sprouts we live-imaged *talin1 crispants* and non-injected controls at 30 hpf and quantified EC number by utilising the endothelial restricted *Tg(fli1ep:nls-mCherry)* marker. Together, this quantitative analysis revealed that EC number was unchanged in *talin1 crispants*, and that thus reduced proliferation is not the cause of shortened ISV sprouts. This new data has now been added to the manuscript as part of **new Supplementary Figure 2 (panels B and C)**.

5. In the DA (Fig 3G)- the cell shape is clearly different, but is there also a difference in cell number in the talin KO?

We agree with the reviewer that, also in the DA, changes in EC number might impact cell rearrangements. We have now re-analysed our data and quantified EC number in the DA of $tln1^{uq1al-/-}$ mutants and siblings at 50 hpf. We utilised VE-cadherin expression from our *TgBAC(ve-cad:ve-cad-TS)* marker line to identify individual ECs. This quantitative analysis revealed that, in agreement with the analysis of EC cell number in the ISVs of *talin1 crispants* **(Supplementary Figure 2 B and C)**, EC number is also not significantly changed in the DA of $tln1^{uq1al-/-}$ mutants. We have now added this new quantitative data to **Figure 3 (panel F)**.

Minor Comments:

A. How do you define biological replicate?

Data analysis from independent clutches of embryos are all considered a "biological replicate". These are either embryos derived from separate paired mating's collected at the same day, or embryos from paired mating's that have been collected on a separate day. We have now simplified the term "biological replicate" to "replicate" in the text.

B. Are the mutant ISVs perfused at 50 hpf before they start regressing? Or are they never perfused at all? Lack of perfusion can contribute to regression so please comment/address within the text.

We thank the reviewer for this interesting question. Our new quantifications of blood flow in $tln1^{uq1al-/-}$ mutants revealed that at 50 hpf the ISVs experience significantly less flow **(new Supplementary Figure 3A)**. Published work however has shown that loss of ISV perfusion does not induce ISV vessel regression[3]. To test whether loss of perfusion induces ISV regression, we treated wild-type embryos with 2,3-butanedione monoxime (BDM) [25mM]. BDM is a muscle relaxant that stops cardiac contraction in zebrafish and is frequently used in the field to temporally stop blood circulation. Embryos were exposed to BDM from 30 hpf till 50 hpf and imaged at the end of treatment. Quantifications of the number of regressed ISVs revealed no significant difference with untreated control embryos. **This new data is added to the manuscript as part of new Supplementary Figure 3 (panels B-C)**.

We have rephrased the concluding statement on page 9 (lines 235-236) to read: "FAs are *likely* to play an essential role in maintaining the vascular network with loss of Talin1 contributing to ISV vessel regression"

© 2022. Published by The Company of Biologists under the terms of the Creative Commons Attribution License (https://creativecommons.org/licenses/by/4.0/).

Reviewer #2: This manuscript by Chau et al., "Dynamically regulated Focal adhesions coordinate endothelial cell remodelling in developing vasculature" is the first to comprehensively examine the effects of focal adhesion defects on vascular morphogenesis and integrity in vivo, in a living organism with intact blood flow. The authors find that the endothelial cells (ECs) of zebrafish talin1 mutants exhibit mature focal adhesion loss, impaired F-actin organization, and reduced inter-EC junction linearization – leading to reduced EC elongation, and angiogenesis and vascular integrity defects. The authors determine that polymerized F-actin is required for EC elongation, and that pharmaceutical stabilization of F-actin partially rescues the EC remodeling and angiogenesis defects of talin1 mutants. Vascular development and integrity defects (including EC actin network deficiency) in zebrafish talin1 mutants have been described previously (Wu et al., 2015). However, this manuscript describes the vascular morphogenesis, focal adhesion, and EC junction defects of talin1 zebrafish mutants in much greater detail, and provides novel mechanistic insights into how focal adhesions and actin cytoskeletal dynamics promote EC elongation and blood vessel maturation in vivo. The authors of this study also develop and introduce a novel transgenic zebrafish model, Tg(fli1ep:Vinculinb-eGFP)^{uq2al}, that can be used to examine EC focal adhesion dynamics in vivo. For these reasons, this manuscript it is of potential significant interest to the vascular development and vascular biology communities.

Overall, the study is interesting, the experiments are generally well performed, the manuscript is well written, and the images and movies are of good quality. However, additional experimentation and clarification is required to address some issues, as described below.

Questions:

1. $tln1^{uq1a1}$ .... is predicted by in silico analysis to disrupt integrin binding. This prediction should be experimentally validated. Are overall Tln1 protein levels changed $tln1^{uq1a1}$-mutants?

As highlighted also by the other reviewers, experimental validation of reduced Talin1 protein levels would strengthen the structural prediction. We now provide **new Western Blot data in Figure 1 (panel D)** of the manuscript, revealing a marked reduction in Talin1 protein expression. Based on this new data we conclude that the in-frame deletion in the FERM domain results in degradation of the mutated protein. We have performed three replicates of this experiment on three independent clutches of embryos. The results for the other two replicates are included here as **Figure 1 for Reviewers**.

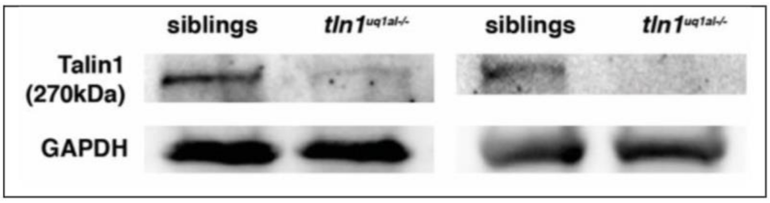

Figure 1: Replicate Western blots for Talin1 (270kDa) in lysates from sibling and $tln1^{uq1al-/-}$ mutant embryos at 3 dpf

2. The authors should determine if EC proliferation or overall migration (e.g., nuclear movement) are altered in the ISVs and dorsal aorta of tln1-mutants.

We thank the reviewer this important question which is in line with Question#4 from Reviewer#1 and below we recap the verification of an additional Talin1 loss of function approach that we established first so that we could address this question:

Since we do not have $tln1^{uq1al}$ fish carrying an endothelial restricted nuclear marker, we established an alternative method of Talin1 loss of function modelling. To do this we adapted a previously reported guideRNA cocktail approach[1].

We designed four individual guideRNAs, all specifically targeting *talin1* genomic sequence. We transcribed and pooled these guides for injection into wild-type embryos at 1-2 cell stage to

© 2022. Published by The Company of Biologists under the terms of the Creative Commons Attribution License (https://creativecommons.org/licenses/by/4.0/).

disrupt Talin1 transcription/translation into functional protein. We further refer to *talin1* guide injected embryos as "*talin1 crispants*".
To validate this approach, we first scored the number of embryos that were phenotypically similar to *tln1*$^{uq1al-/-}$ stable mutants at 50 hpf. Upon two independent injection rounds we determined that ~80% of *talin1 crispants* were indistinguishable from *tln1*$^{uq1al-/-}$ mutants, developing bleedings in the brain and mild cardiac oedema. The remaining 20% of embryos were either less severe, characterised by focal bleedings in the brain without prominent cardiac phenotypes, or more severe, developing severe cardiac oedema and complete loss of circulation.
We next determined DNA cutting efficiency for each of the four guides by amplifying the targeted amplicon. We performed this analysis on 8 injected embryos form each of the two independent injection rounds and identified that each guide induced an overall lack of a distinctive amplicon PCR band and the appearance of additional amplicons of different sizes. These results indicated CRISPR/Cas9 induced changes to the genomic region targeted by these guideRNAs.
This extensive validation has been added to **Supplementary Figure 1 (panels D-F)**.

To examine EC number in the ISVs, we live-imaged *talin1 crispants* and non-injected controls at 30 hpf and quantified EC number by utilising the endothelial restricted *Tg(fli1ep:nls-mCherry)* marker. Together, this quantitative analysis revealed that EC number was unchanged in *talin1 crispants*, and that thus reduced proliferation is not the cause of shortened ISV sprouts. This **new data has been added to the manuscript as part of new Supplementary Figure 2 (panels B-C).**
Related to this question and in response to reviewer#1, we also found that in the DA at 50 hpf the number of ECs is also not significantly different. This analysis was done by analysing stable *tln1*$^{uq1al-/-}$ mutants and has been added as new quantitative data to
Figure 3 (panel F).

3. To further substantiate their claim that Talin1 functions EC autonomously, the authors should determine either (i) if the ECs of wild type donor embryos elongate properly when transplanted into tln1-mutant recipients, or (ii), if expressing wild type talin1 specifically in the ECs of tln1-mutants rescues their EC-elongation and/or ISV angiogenesis defects.

We thank the reviewer for raising the important issue of cell-autonomy. We agree that complementary experiments can strengthen the observation that Talin1 acts cell- autonomously.
However, we decided not to analyse wild-type ECs in *tln1*$^{uq1al-/-}$ mutant animals (suggestion i by reviewer), since we know that flow pressure will be fully lost in mutant animals at 3 dpf which could skew the analysis of transplanted wild-type cells at this developmental stage.
We agree that EC-restricted expression of wild-type *talin1* in *tln1*$^{uq1al-/-}$ ECs could have been informative (suggestion ii by reviewer), however we believe this can result in overexpression phenotypes, as we have experienced previously when mosaically injecting BAC DNA expressing VE-cadherin[4].

To address the issue of cell-autonomy more specifically, we have instead performed additional transplantation experiments of Talin1 deficient ECs into wild-type embryos. In these new experiments we have visualised all transplanted cells (not just ECs), by injecting the donors with cascade blue dye at the 1-cell stage. We next transplanted cells from double transgenic *talin1 crispant* donors into wild-type recipients. The donors expressed both *TgBAC(ve-cad:ve-cad-TS)*, labelling VE-cadherin at cell-cell junctions and *Tg(fli1ep:nls-mCherry),* marking the EC nuclei. Talin1 deficient donor cells were transplanted into *TgBAC(ve-cad:ve-cad-TS)* wild-type recipients. This allowed us to visualise and quantify EC shape of both Talin1 deficient donor ECs and neighbouring wild-type ECs.
We identified EC restricted grafts in n=8 embryos from 2 independent rounds of transplantations. We quantified EC shape of Talin1 deficient donor cells versus neighbouring wild-type ECs at 2 and 3 dpf. This analysis confirmed our previous findings that Talin1 is required cell-autonomously for EC elongation. This **new data is added to the manuscript in Figure 4 (panels E-G).**

Minor Comments:
A. The authors should determine if brain vascular development is also perturbed in tln1-mutants.
We thank the reviewer for suggesting this more detailed analysis of brain vasculature development.

© 2022. Published by The Company of Biologists under the terms of the Creative Commons Attribution License (https://creativecommons.org/licenses/by/4.0/).

We have now performed live imaging of both $tln1^{uq1al-/-}$ mutants and siblings at 48 hpf. This analysis revealed that the cerebral blood vessel network was less complex in $tln1^{uq1al-/-}$ mutants and that major vessels either did not form or had regressed at this stage. This data has now been added to the manuscript as part of **Supplementary Figure 1 (panel B).**

B. In embryos transplanted with tln1-mutant cells, do neighboring wild type recipient ECs also share this phenotype? This should be examined.
We would like to refer to our response to question #3 above. Our new transplant data shows that neighbouring wild-type cells are significantly more elongated compared to Talin1 deficient EC clones both at 2 dpf and 3 dpf. **This new data has been added to the manuscript as part of Figure 4 (panels E-G).**

C. Given that EC elongation and junction linearity in tln1-mutants is partially rescued following Jasplakinolide treatment (actin stabilization), the authors should indicate if the hemorrhage phenotype is also rescued.

We thank the reviewer for suggesting this intriguing rescue experiment. We treated both $tln1^{uq1al-/-}$ mutants and siblings with either 0.1% DMSO or Jasplakinolide [1µM] from 24 to 50 hpf and scored the number of embryos with bleedings in the brain upon treatment. This analysis revealed that Jasplakinolide did not significantly rescue bleedings in the brain. The percentage of $tln1^{uq1al-/-}$ mutant embryos developing cranial haemorrhaging when treated with DMSO was 62.5%, compared to 57% in Jasplakinolide treated mutants. Jasplakinolide treatment of sibling embryos had no adverse effects and embryos were undistinguishable from DMSO treated sibling embryos.
This data, together with the new images of the cerebral vasculature at 48 hpf **(Supplemental Figure 1B),** suggests that FAs likely control a more complex series of events that are essential for the formation, integrity, and maintenance of the highly specialised cerebral vasculature that are not corrected by stabilisation and expansion of polymerised F-actin. The focus of our manuscript has been on EC elongation of in the more moderately affected trunk vasculature, since this allowed us to investigate FA- deficient ECs in flow pressured vasculature without such major disruptions to network perfusion. We have therefore decided to not include this data as part of the revised manuscript, but we have added the results here as **Figure 3 for reviewers**.

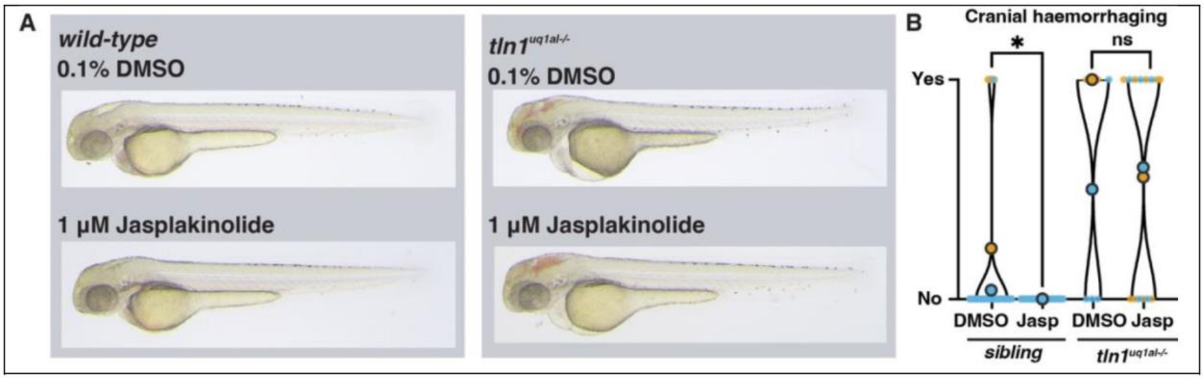

Figure 3: (A) Left: Representative brightfield images of wild-type embryos treated with 0.1% DMSO or Jasplakinolide [1µM]. Right: Representative brightfield images of $tln1^{uq1al-/-}$ mutant embryos treated with 0.1% DMSO or Jasplakinolide [1µM], both developing with cranial haemorrhaging. (B) Scoring of the presence or absence of cranial haemorrhaging phenotype in $tln1^{uq1al-/-}$ mutants and siblings at 50 hpf, revealing Jasplakinolide does not rescue this phenotype. Graphs present n=2 replicates, n=90 siblings and n=22 $tln1^{uq1al-/-}$ mutants. Replicate averages are depicted by large circles and smaller circles present individual data points of each replicate (colour matched).

Reviewer #3: Chau and colleagues analyze the requirement of Talin-1 during blood vessel formation and remodeling during early zebrafish development. To this end, they generate a targeted mutation of talin-1. They also generate a transgenic reporter line to visualize Vinculin in endothelial cells. Using these tools the authors show that talin-1 mutant lack focal adhesions.

© 2022. Published by The Company of Biologists under the terms of the Creative Commons Attribution License (https://creativecommons.org/licenses/by/4.0/).

They further show that Talin-1 is required for efficient sprouting, junctional rearrangement during ISV formation, blood vessel maintenance and endothelial cell elongation. Analysis of genetic mosaics demonstrates that Talin-1 is required cell-autonomously. Finally, to identify the cellular mechanisms which are affected in talin-1 mutants, they image F-actin and manipulate F-actin dynamics by pharmacological intervention. Whereas, inhibition of Arp2/3 mimics aspects of the talin1 mutant phenotype, stabilization of F-actin fibers by treatment with jasplakinolide can rescue the mutant phenotype. Overall, the authors use a new model to study focal adhesions during vascular morphogenesis and present a set of very interesting findings linking focal adhesions to junctional dynamics underlying vascular tube formation.

Questions:

1. The authors suggest that the generated talin-1 mutant is a null allele, but do not present data that would support this view.
A potential dominant-negative effect could be determined by comparing wild-type, heterozygous and homozygous talin-1 mutants.

We thank the reviewer for this question. We agree that it is important to clearly establish the effect of the in-frame deletion upon Talin1 protein abundance. We now provide **new Western Blot data in Figure 1 (panel D) of the manuscript,** revealing a marked reduction in Talin1 protein expression. Based on this new data we conclude that the in- frame deletion in the FERM domain results in degradation of the mutated protein. We have performed three replicates of this experiment on three independent clutches of embryos. The results for the replicates are shown here as **Figure 1 for Reviewers**.

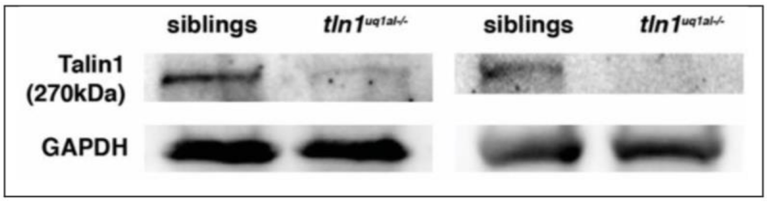

Figure 1: Replicate Western blots for Talin1 (270kDa) in lysates from sibling and *tln1*$^{uq1al-/-}$ mutant embryos at 3
dpf

2. The presence of talin paralogs raises the issue of functional redundancy. Do the authors have any information on the expression patterns of the paralogs?
Later on the authors mention that maternal talin-1 contribution may partially rescue loss of zygotic Talin-1. Do the authors have concrete information on maternal talin-1 RNA or protein. Are talin-1 mutants homozygous viable/fertile?

We thank the reviewer for requesting a more detailed explanation with regards to *talin* paralogs and maternal contribution of *talin1*.

First, extensive *in situ* hybridisation experiments have been performed by other previously[5]. This analysis revealed the spatio-temporal expression patterns of *talin* paralogs in zebrafish[5]. This data shows that *talin1* is the main paralog expressed in cells of the heart and the vasculature. These published results combined with our qRT-PCR data **(Supplementary Figure 1G)**, suggest that functional redundancy by paralogs is less likely to occur.

We further thank the reviewer for prompting us to substantiate our hypothesis that *talin1* could be maternally contributed. To examine if *talin1* mRNA was present in embryos prior to maternal-zygotic transition, we performed a RT-PCR time-course. We generated cDNA from a pool of 20 wild-type embryos at 1-cell stage, 1 hpf, 3 hpf, 1 dpf and 2 dpf and examined *talin1* mRNA expression in these samples. We included mRNA expression analysis of control genes that were verified in by Harvey et al[6], including; *cyclinb1* (maternally provided), C18H16orf7 (maternally provided, but not transcribed after zygotic transition) and *tal1* (not maternally provided). This analysis indisputably showed that *talin1* mRNA is indeed maternally provided, supporting our hypothesis that this

© 2022. Published by The Company of Biologists under the terms of the Creative Commons Attribution License (https://creativecommons.org/licenses/by/4.0/).

contribution would partially compensate for Talin1 loss early in development. We have added this **new data in Supplementary Figure 2 (panel A)**.

Finally, to answer the reviewer's question with regards to the viability of *tln1*$^{uq1al-/-}$ mutants, we would like to emphasize that homozygous *tln1*$^{uq1al-/-}$ mutants are not viable. In fact, the overall phenotype of *tln1*$^{uq1al-/-}$ mutants worsens rapidly after 3 dpf. To visualise the correlation between genotype and phenotype we have taken a series of brightfield images of *tln1*$^{uq1al-/-}$ mutants and siblings at both 2 dpf and 5 dpf, presented in **Figure 2 for reviewers** below. This data shows that heterozygotes are indistinguishable from homozygous wild-type animals. However, the *tln1*$^{uq1al-/-}$ mutation is lethal and embryos are severely compromised and not viable beyond 5 dpf.

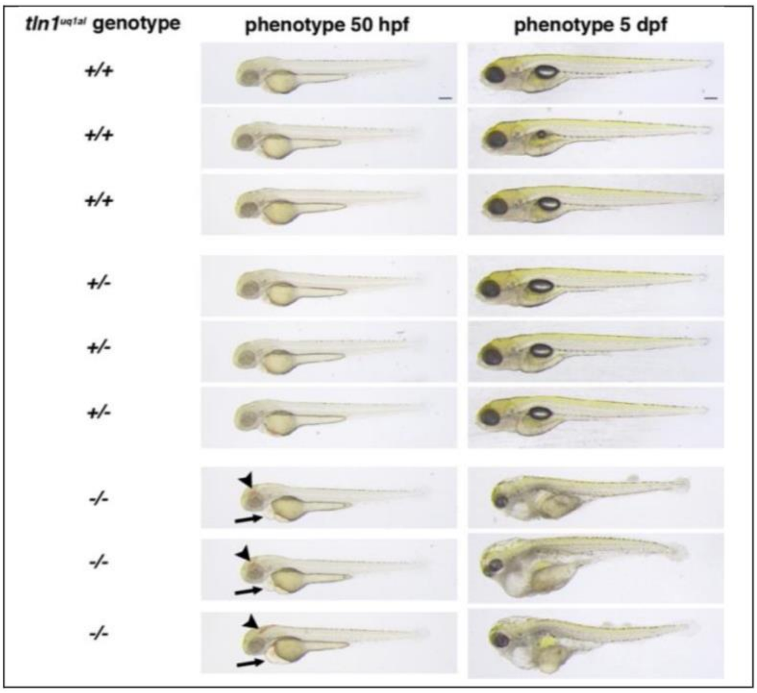

Figure 2: Representative brightfield images of each *tln*^{uq1al} genotype (3 embryos per genotype are shown). Homozygous wild-type (top) embryos are indistinguishable from heterozygotes (middle) at both 2 dpf and 5 dpf, whilst homozygous mutant embryos present with cranial haemorrhages (arrowheads) and cardiac edema (arrows) at 2 dpf. At 5 dpf, the overall phenotype of homozygous mutants has worsened, and embryos are not viable past this developmental stage. Scale bar = 100µm

3. The loss of Vinculin-GFP clusters in talin-1 mutants is quite dramatic. It would be important to have a second independent to demonstrate that these clusters are indeed focal adhesions. The authors should try antibodies against FAK and P- Paxillin, which have previously been shown to work in heart of zebrafish embryos.

We thank the reviewer for this comment, and we agree that an additional marker would strengthen our observations. To address this question, we have now tested a cohort of 8 antibodies for FA proteins, including those suggested by the reviewer from Gunawan et al[7]. However, we did not detect, what we would consider, specific expression at FA structures in the vasculature when using any of our standardised IF protocols[8-11]. Notably, we did observe specific staining at muscle boundaries for n=6 of these antibodies. This suggested to us that these antibodies do indeed cross react with zebrafish proteins but fail to detect FAs in the vasculature.

To our knowledge, other studies do not exist that have positively localised endogenous FA proteins in the zebrafish vasculature. We therefore hypothesised that endothelial FAs deteriorate or detach

© 2022. Published by The Company of Biologists under the terms of the Creative Commons Attribution License (https://creativecommons.org/licenses/by/4.0/).

during whole mount fixation. Extensive trouble shooting of the methodology led us to develop a new approach to fix ECs more directly. We performed intra-venous injections of 4% paraformaldehyde into the blood stream of anaesthetised embryos, followed by whole mount fixation. Using this method, we identified that Vinculinb-eGFP indeed co-localises with pPaxillin at FAs in wild-type embryos. In *tln1*$^{uq1al-/-}$ mutants, significantly less pPaxillin positive FAs could be observed, verifying our observations by live imaging of Vinculinb-eGFP **(Figure 1E,F)**. The new immunofluorescence data has been added here as **Figure 4 for reviewers**.
We would like to note that this new fixation method requires some further optimisation since we could not detect prominent Vinculinb-eGFP or pPaxillin staining in the DA. The methodology also did not work as effectively in all injected embryos. Due to this variability, we did not quantify changes in pPaxillin expression in mutants. We have added the co-localisation data in wild-types to the manuscript in **Supplementary Figure 1 (panel H)**. Since other zebrafish vascular biologists are likely facing similar issues when trying to positively identify FAs in vasculature, we are now further optimising this method and plan to publish this in the future as a technical report.

To provide the reviewer with a more extensive overview of Vinculinb-eGFP expression that we observe in *tln1*$^{uq1al-/-}$ mutants, we have included additional examples of Vinculinb- eGFP expression in both *tln1*$^{uq1al-/-}$ mutants and siblings (**Figure 5 for reviewers**)

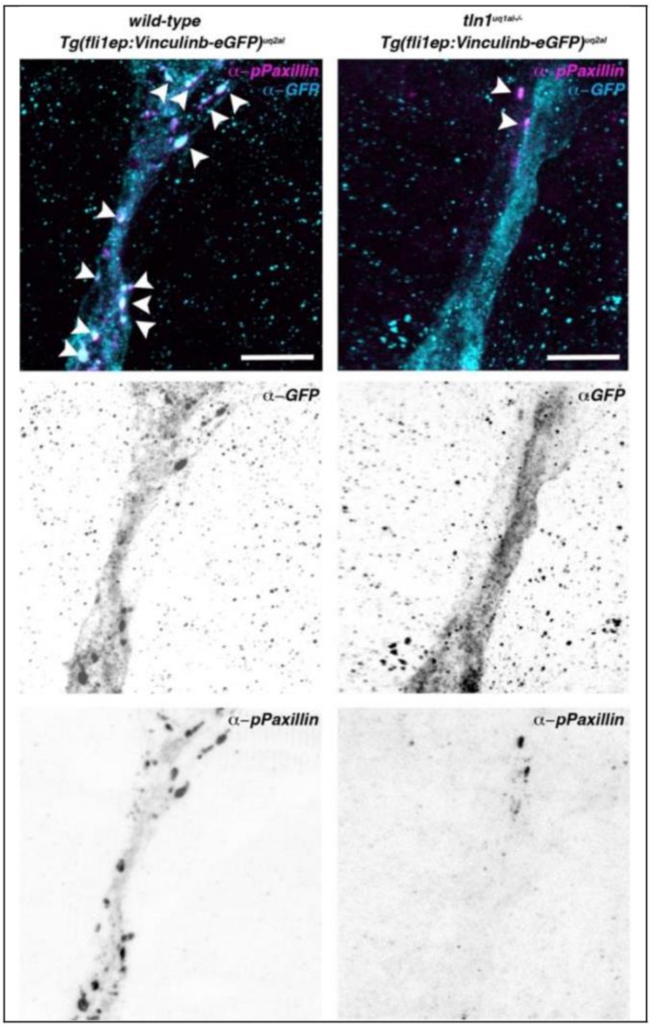

Figure 4: Maximum intensity projection of intersegmental vessels (ISVs) in a wild- type (left) and *tln1*$^{uq1al-/-}$ mutant embryo (right), both carrying the *Tg(fli1ep:Vinculinb-eGFP)*^{uq2al} transgene. Blood vessels were fixed by intravenous injections of 4%PFA at 50 hpf, followed by whole mount fixation. Fixed embryos were stained for Vinculinb-eGFP using an α-GFP antibody and for the FA marker pPaxillin (pPax-Y118). White arrowheads indicate co-localisation in wild-type ISVs. Scale bar = 10μm

© 2022. Published by The Company of Biologists under the terms of the Creative Commons Attribution License (https://creativecommons.org/licenses/by/4.0/).

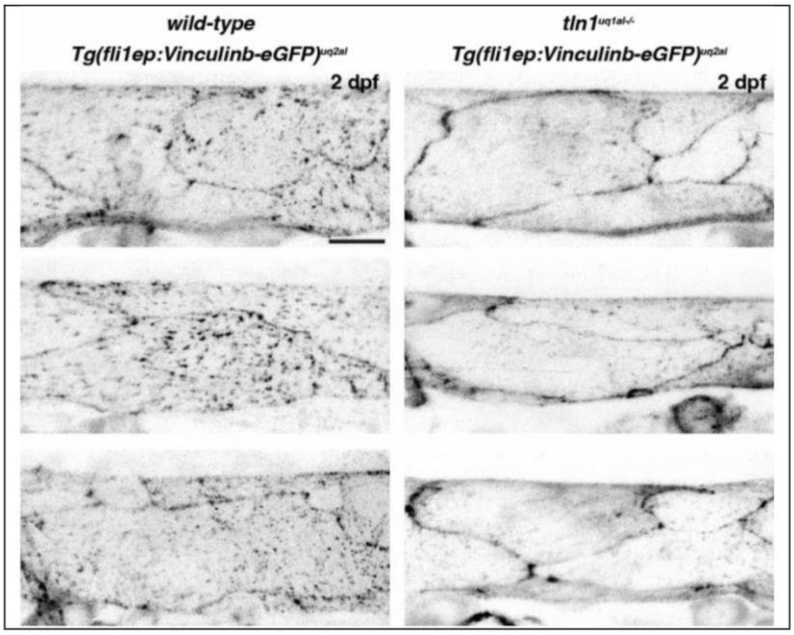

Figure 5: Three examples of Vinculinb-eGFP expression in the dorsal aorta of both wild- type embryos (left) and *tln1*$^{uq1al-/-}$ mutants (right). Similar to representative embryos presented in Figure 1D, Vinculin-eGFP is vastly reduced at FA clusters but still localises to cell-cell junctions in *tln1*$^{uq1al-/-}$ mutants.

4. Figure 1: Do the authors see similar changes with junctional markers such as the VE-Cad-TS transgene? Panel G: Is the cell-cell junction narrowed in a comparable fashion in the VE-cad-TS line?

We thank the reviewer for suggesting further quantitation of our VE-cadherin data. We have now re-analysed our data and quantified the width of VE-cadherin junctions in both *tln1*$^{uq1al-/-}$ mutants and siblings at 50 hpf. This analysis did not reveal a significant difference in junction width when Talin1 is lost. This **new quantified data has been included to Figure 3 (panel H).**

5. As filopodia are very dynamic cellular processes, it would be important to perform time-lapse imaging to determine, whether filopodial dynamics are disturbed in talin-1 mutants.

We agree with the reviewer that even though filopodia number and length is not changed in single confocal stacks, Talin1 might still impact the dynamic behaviour of these filopodia. To analyse filopodia dynamics we performed high resolution time-lapse imaging at 26 hpf using the membranous *Tg(kdr-l:Hsa.HRAS-mCherry)*^{s916} line which faithfully labels filopodial extensions[12]. Quantitative analysis of filopodia dynamics revealed that there is a significant reduction in the lifetime of Talin1 deficient filopodia. Whilst filopodia persist in wild-type embryos during the time course of the movies, in *tln1*$^{uq1al-/-}$ mutants filopodia retract more quickly. These results suggest that anchoring of filopodia to the ECM is compromised leading to retraction of the filopodia. We believe that these unstable filopodia are likely to contribute to reduced ISV sprouting efficiency in *tln1*$^{uq1al-/-}$ mutants since filopodia are known to assist in sprouting[12]. This new data has been added to the manuscript as part of **Figure 2 (panel F) and as a new Supplementary Movie 2.**

6. Could the defects in DLAV remodeling be caused by lack of blood flow in the ISV/DLAV? Did the authors check for blood flow in these vessels?
Is the disconnection within the ISV preceded by loss of blood flow?

We agree with the reviewer that reduced DLAV plexus formation might be a consequence of flow loss. In response to Reviewer 1 (Question#2), we performed quantifications of blood flow velocity in the DA and ISVs at 50 hpf. This analysis revealed that there was a significant reduction of flow in

© 2022. Published by The Company of Biologists under the terms of the Creative Commons Attribution License (https://creativecommons.org/licenses/by/4.0/).

ISVs at this stage, whilst flow in the DA is not significantly changed. This **new analysis is part of new Supplementary Figure 3 (panel A) and has been added to the manuscript as Movies 4 and 5**. This data suggests that reduced flow might indeed contribute to compromised DLAV plexus formation in *tln1^{uq1al}* mutants.

To directly assess if blood flow is required for DLAV plexus formation, independent of Talin1, we treated wild-type embryos with 2,3-butanedione monoxime (BDM) [25mM]. BDM is a muscle relaxant that stops cardiac contraction in zebrafish and is frequently used in the field to temporally stop blood circulation. Embryos were exposed to BDM during DLAV plexus formation, from 30 hpf till 50 hpf and imaged at the end of treatment. We found that DLAV plexus formation was strongly compromised in BDM treated embryos. This **new data has been added to Supplementary Figure 3 (panel B).**
We conclude form this data that blood flow is indeed required for DLAV plexus formation and that reduced flow in ISVs of *tln1$^{uq1al-/-}$* mutants likely contributes to the loss of DLAV formation.
We did not observe enhanced ISV regression in BDM treated embryos, showing that vessel regression is not induced by flow loss. This is supported by published work[3] describing a zebrafish *endoglin* mutant wherein stochastic loss of ISV perfusion also does not induce ISV vessel regression.

7. From the data shown, it appears that many of the defects observed in talin-1 mutants may ultimately be caused by junctional destabilization. Since this group has established the VE-cadherin-based tension sensor in zebrafish, I wonder whether they have examined junctional tension in talin-1 mutant.

We thank the reviewer for pointing out this valuable added utility of the VE-cadherin transgenic line that we published in 2017[4]. After our identification of cell elongation defects and actin defects in *tln1$^{uq1al-/-}$* mutants, we have attempted FRET based tension measurements in 2019-2020. The main hurdle over these past few years has been the decline of laser power on the confocal microscope that is suitable to image Teal fluorescent protein at our institute. This decline in laser capacity has resulted in a signal- to-noise-ratio that we have determined to be unacceptable for corrected FRET measurements. There are no other microscopes available with the imaging capabilities we require for such FRET measurements.
To circumvent these imaging issues, we are currently developing a new VE-cadherin tension sensor line whereby we have replaced Teal and Venus with a mNeonGreen and mScarlet-I as the FRET pair in the tension sensor module. mNeonGreen-mScarlet-I have been validated recently to be extremely suitable as a FRET pair and show much improved brightness and quantum yield[13]. We anticipate that once this new transgenic line is established, this will vastly improve feasibility of FRET measurements.

8. Figure 6: The interpretation of the jasplakinolide may not be as straight forward as the authors suggest. Besides stabilizing F-actin fibers, it has been shown to enhance F-actin nucleation and polymerization in cell culture (e.g. Bubb et al., Journal of Biological Chemistry (2000)). Therefore, it is possible (or even more likely) that the phenotypic rescue of talin-1 mutants by jasplakinolide is mediated by enhanced F-actin polymerization.

We completely agree with the reviewer that enhanced actin polymerisation is also expected to contribute to the phenotypic rescue. In response to their comment of the reviewer we have now re-phrased the description of Jasplakinolide in the text on page 12 (lines 357-359) to: "*we utilised Jasplakinolide (Jasp), a compound which stabilises actin filaments by reducing disassembly and promotes polymerisation through F-actin nucleation*[14,15]*.*"

We have further changed our description of the results on page 13 (line 374-376) to: "*Taken together, these experiments show that enhancing F-actin polymerisation and promoting the stability of these networks can result in sufficient acto-myosin activity that is required to elongate FA-deficient ECs and for junctions to linearise. Whether compensation occurs via few remaining FAs (Fig. 1E), or whether elongation is mainly driven by JBLs at cell-cell junctions remains to be determined.*"

© 2022. Published by The Company of Biologists under the terms of the Creative Commons Attribution License (https://creativecommons.org/licenses/by/4.0/).

Minor points:

1. "To our knowledge, this represents the first in vivo model that reports FA dynamics in ECs of live vasculature"(p8) This statement is more appropriate for the Discussion section.

We agree with the reviewer and have moved this statement from the results section to the discussion on page 14 (lines 387-390). Specifically, it is explained as follows: *"To our knowledge, this Vinculin transgenic line represents the first in vivo model that reports FA dynamics in ECs of live vasculature and therefore will provide a unique resource to the cardiovascular research community for future studies into FA function."*

2. "… anchorage of filopodia to the ECM occurs via FAs ". (p8) This statement should be referenced

We thank the reviewer for pointing this out. We have now referenced a suitable review article.

3. " … EC elongation is involved in the transformation of pre-mature unicellular ISVs …" (p9) unicellular ISVs are not pre-mature.

In response to the reviewer's comment, we have removed the wording "pre-mature" in the manuscript when describing unicellular ISVs.

4. References 43 (Bentley) 44 (Perryn) do not support the statement about multicellular tube formation.

We thank the reviewer for identifying this oversight. We have removed these references when describing multicellular tube formation on page 9.

5. What do the authors mean by "Guided" EC elongation?

Guided in this context referred to the FAs being essential for the process of EC elongation. We understand that this wording can be confusing, and we thank the reviewer for pointing this out. We have now removed the word "guided" in this revised version of the manuscript.

6. "FAs are essential to facilitate EC elongation" (p10/11)- I am not sure what the authors mean by "facilitate".

"Facilitate" meant to describe that EC elongation does not occur to the same degree as it does in wild-type embryos when FAs are lost. We have replaced facilitate with "required" or "essential" throughout this revised version of manuscript to clarify this.

REFERENCES:

1 Wu, R. S. *et al*. A Rapid Method for Directed Gene Knockout for Screening in G0 Zebrafish. *Dev Cell* **46**, 112-125 e114, doi:10.1016/j.devcel.2018.06.003 (2018).

2 Vignes, H. *et al*. Extracellular mechanical forces drive endocardial cell volume decrease during zebrafish cardiac valve morphogenesis. *Dev Cell* **57**, 598-609 e595, doi:10.1016/j.devcel.2022.02.011 (2022).

3 Sugden, W. W. *et al*. Endoglin controls blood vessel diameter through endothelial cell shape changes in response to haemodynamic cues. *Nat Cell Biol* **19**, 653-665, doi:10.1038/ncb3528 (2017).

4 Lagendijk, A. K. *et al*. Live imaging molecular changes in junctional tension upon VE-cadherin in zebrafish. *Nat Commun* **8**, 1402, doi:10.1038/s41467-017-01325- 6 (2017).

5 Wu, Q. *et al*. Talin1 is required for cardiac Z-disk stabilization and endothelial integrity in zebrafish. *FASEB J* **29**, 4989-5005, doi:10.1096/fj.15-273409 (2015).

6 Harvey, S. A. *et al*. Identification of the zebrafish maternal and paternal transcriptomes. *Development* **140**, 2703-2710, doi:10.1242/dev.095091 (2013).

7 Gunawan, F. *et al*. Focal adhesions are essential to drive zebrafish heart valve morphogenesis. *J Cell Biol* **218**, 1039-1054, doi:10.1083/jcb.201807175 (2019).

8 Koltowska, K. *et al*. The RNA helicase Ddx21 controls Vegfc-driven developmental lymphangiogenesis by balancing endothelial cell ribosome biogenesis and p53 function. *Nat Cell Biol* **23**, 1136-1147, doi:10.1038/s41556-021-00784-w (2021).

9 Baek, S. *et al*. The Alternative Splicing Regulator Nova2 Constrains Vascular Erk Signaling to Limit Specification of the Lymphatic Lineage. *Dev Cell* **49**, 279-292 e275, doi:10.1016/j.devcel.2019.03.017 (2019).

10 De Angelis, J. E. *et al*. Tmem2 Regulates Embryonic Vegf Signaling by Controlling Hyaluronic

© 2022. Published by The Company of Biologists under the terms of the Creative Commons Attribution License (https://creativecommons.org/licenses/by/4.0/).

Acid Turnover. *Dev Cell* **40**, 123-136, doi:10.1016/j.devcel.2016.12.017 (2017).
11 Koltowska, K. *et al*. Vegfc Regulates Bipotential Precursor Division and Prox1 Expression to Promote Lymphatic Identity in Zebrafish. *Cell Rep* **13**, 1828-1841, doi:10.1016/j.celrep.2015.10.055 (2015).
12 Phng, L. K., Stanchi, F. & Gerhardt, H. Filopodia are dispensable for endothelial tip cell guidance. *Development* **140**, 4031-4040, doi:10.1242/dev.097352 (2013).
13 McCullock, T. W., MacLean, D. M. & Kammermeier, P. J. Comparing the performance of mScarlet-I, mRuby3, and mCherry as FRET acceptors for mNeonGreen. *PLoS One* **15**, e0219886, doi:10.1371/journal.pone.0219886 (2020).
14 Holzinger, A. Jasplakinolide: an actin-specific reagent that promotes actin polymerization. *Methods Mol Biol* **586**, 71-87, doi:10.1007/978-1-60761-376-3_4 (2009).
15 Bubb, M. R., Spector, I., Beyer, B. B. & Fosen, K. M. Effects of jasplakinolide on the kinetics of actin polymerization. An explanation for certain in vivo observations. *J Biol Chem* **275**, 5163-5170, doi:10.1074/jbc.275.7.5163 (2000).

Second decision letter

MS ID#: DEVELOP/2021/200454

MS TITLE: Dynamically regulated Focal adhesions coordinate endothelial cell remodelling in developing vasculature

AUTHORS: Chui-Ying (Tevin) Chau, Mikaela Sarah Keyser, Jason A Da Silva, Elysse Kiara Morris, Teodor E Yordanov, Kinga P Duszyc, Scott Paterson, Alpha Yap, Benjamin M Hogan, and Anne Karine Lagendijk

You will be pleased that the reviewers like your revised manuscript and there are just a couple of very minor issues to consider before we proceed to publication. The referees' comments are appended below, or you can access them online: please go to BenchPress and click on the 'Manuscripts with Decisions' queue in the Author Area.

Reviewer 1

Advance summary and potential significance to field

n/a

Comments for the author

The authors have done a wonderful job of addressing all of my concerns. Congrats on a beautiful manuscript.

Reviewer 2

Advance summary and potential significance to field

This manuscript by Chau et al., “Dynamically regulated Focal adhesions coordinate endothelial cell remodelling in developing vasculature” is the first to comprehensively examine the effects of focal adhesion defects on vascular morphogenesis and integrity in vivo, in a living organism with intact blood flow.
The authors find that the endothelial cells (ECs) of zebrafish talin1 mutants exhibit mature focal adhesion loss, impaired F-actin organization, and reduced inter-EC junction linearization – leading to reduced EC elongation, and angiogenesis and vascular integrity defects. The authors determine that polymerized F-actin is required for EC elongation, and that pharmaceutical stabilization of F-actin partially rescues the EC remodeling and angiogenesis defects of talin1 mutants.

© 2022. Published by The Company of Biologists under the terms of the Creative Commons Attribution License (https://creativecommons.org/licenses/by/4.0/).

Vascular development and integrity defects (including EC actin network deficiency) in zebrafish talin1 mutants have been described previously (Wu et al., 2015).
However, this manuscript describes the vascular morphogenesis, focal adhesion, and EC junction defects of talin1 zebrafish mutants in much greater detail, and provides novel mechanistic insights into how focal adhesions and actin cytoskeletal dynamics promote EC elongation and blood vessel maturation in vivo.
The authors of this study also develop and introduce a novel transgenic zebrafish model, Tg(fli1ep:Vinculinb-eGFP)uq2al, that can be used to examine EC focal adhesion dynamics in vivo.
For these reasons, this manuscript is of significant interest to the vascular development and vascular biology communities.

Comments for the author

The authors have done a great job in revising the manuscript and have addressed my comments satisfactorily. It is a fantastic contribution to the field.

Extremely minor points:
The sentences in lines 111 to 117 of the introduction are either identical, or nearly identical to lines 119 – 123. I would advise removing the duplicated text.
Beginning at line 463 in the Materials and Methods section, some of the " ' " symbols appear as boxed question marks in the pdf document. This is also true beginning at line 499.

Reviewer 3

Advance summary and potential significance to field

In this study Chau and coworkers present a new genetic model to study the role of focal adhesions (FAs) in blood vessel morphogenesis. Although FAs have been extensively studied in in vitro studies, in vivo studies are lacking - in particular with respect to how FAs contribute to the cellular behaviors and dynamics underlying blood vessel formation and dynamic cell shape changes.
The present study presents novel insights into this subject. Overall, this is an in-depth phenotypic analysis, using novel tools and thorough experimentation. It will be of great interest to the developmental and cell biology research community.

Comments for the author

The authors have invested a considerable amount of work and present new data which greatly improve the quality of this study. All my concerns have been addressed.
1) With respect to the potential maternal contribution, the authors convincingly show that talin-1 is maternally expressed. I wonder, whether the authors have tested the relevance of this by blocking translation (ATG-MOs) of the maternal mRNA.
2) The term "junctional linearity" is not really explained - a short definition would be helpful.

Second revision

Author response to reviewers' comments

Response to Reviewer's comments

We thank all reviewers for their positive response to our revised version of the manuscript. Below we have outlined our response to the minor questions/ comments from reviewer #2 and #3.

Reviewer #2:

Questions:

1. The sentences in lines 111 to 117 of the introduction are either identical, or nearly

© 2022. Published by The Company of Biologists under the terms of the Creative Commons Attribution License (https://creativecommons.org/licenses/by/4.0/).

identical to lines 119 - 123. I would advise removing the duplicated text.
We thank the reviewer for pointing this out. We agree that these sections of the text were repetitive. We have removed the following text: *"In this study, we sought to better understand the early vascular impact of FAs, using a novel zebrafish talin1 mutant model, tln1^{uq1al}. In tln1$^{uq1al-/-}$ mutants cardiac output remains stable until two days post fertilisation (2 dpf), and thus blood circulation persists, providing a unique opportunity to study the EC-specific consequences of compromised FA function in flow pressured vasculature."*

2. Beginning at line 463 in the Materials and Methods section, some of the " ' " symbols appear as boxed question marks in the pdf document. This is also true beginning at line 499.
When converting our Word file of the manuscript to a pdf we have not identified this issue. We will ensure to verify symbols are properly displayed prior to publication.

Reviewer #3:

Questions:

1. With respect to the potential maternal contribution, the authors convincingly show that talin-1 is maternally expressed. I wonder, whether the authors have tested the relevance of this by blocking translation (ATG-MOs) of the maternal mRNA.
We thank the reviewer for their interest in the function of maternally contributed Talin1. We have not used any ATG-MOs, but instead we are generating maternal-zygotic (MZ) *tln1$^{uq1al-/-}$* mutants by germ cell transplantation. We will analyse the phenotypic consequences of maternal Talin1 depletion as part of another project in the lab.

2. The term "junctional linearity" is not really explained - a short definition would be helpful.
We appreciate that explanatory text on junctional linearity would improve clarity of this phenomenon for readers. We have now included the following text on page 9-10, lines 256-258: *"An additional hallmark of EC remodelling in the DA is linearisation of EC junctions, whereby irregular junctions straighten over time (Lagendijk et al., 2017)."*
We have further added the following description of how junctional linearity is quantified on page 21, lines 628-630: *"Junctional linearity: The junctional linearity index was calculated by dividing the distance between junctional vertices over the length of the junction (Lagendijk et al., 2017). The closer to 1 in the index, the straighter the junction.*

<u>Third decision letter</u>

MS ID#: DEVELOP/2021/200454

MS TITLE: Dynamically regulated Focal adhesions coordinate endothelial cell remodelling in developing vasculature

AUTHORS: Chui-Ying (Tevin) Chau, Mikaela Sarah Keyser, Jason A Da Silva, Elysse Kiara Morris, Teodor E Yordanov, Kinga P Duszyc, Scott Paterson, Alpha Yap, Benjamin M Hogan, and Anne Karine Lagendijk
ARTICLE TYPE: Research Article

I am happy to tell you that your manuscript has been accepted for publication in Development, pending our standard ethics checks.

© 2022. Published by The Company of Biologists under the terms of the Creative Commons Attribution License (https://creativecommons.org/licenses/by/4.0/).
